# Supplementary figures and images for: Aminoglycoside tolerance in Vibrio cholerae engages translational reprogramming associated with queuosine tRNA modification
Source: eLife. 2025 Jan 6;13:RP96317. doi: 10.7554/eLife.96317 (PMC11703503; doi:10.7554/eLife.96317)

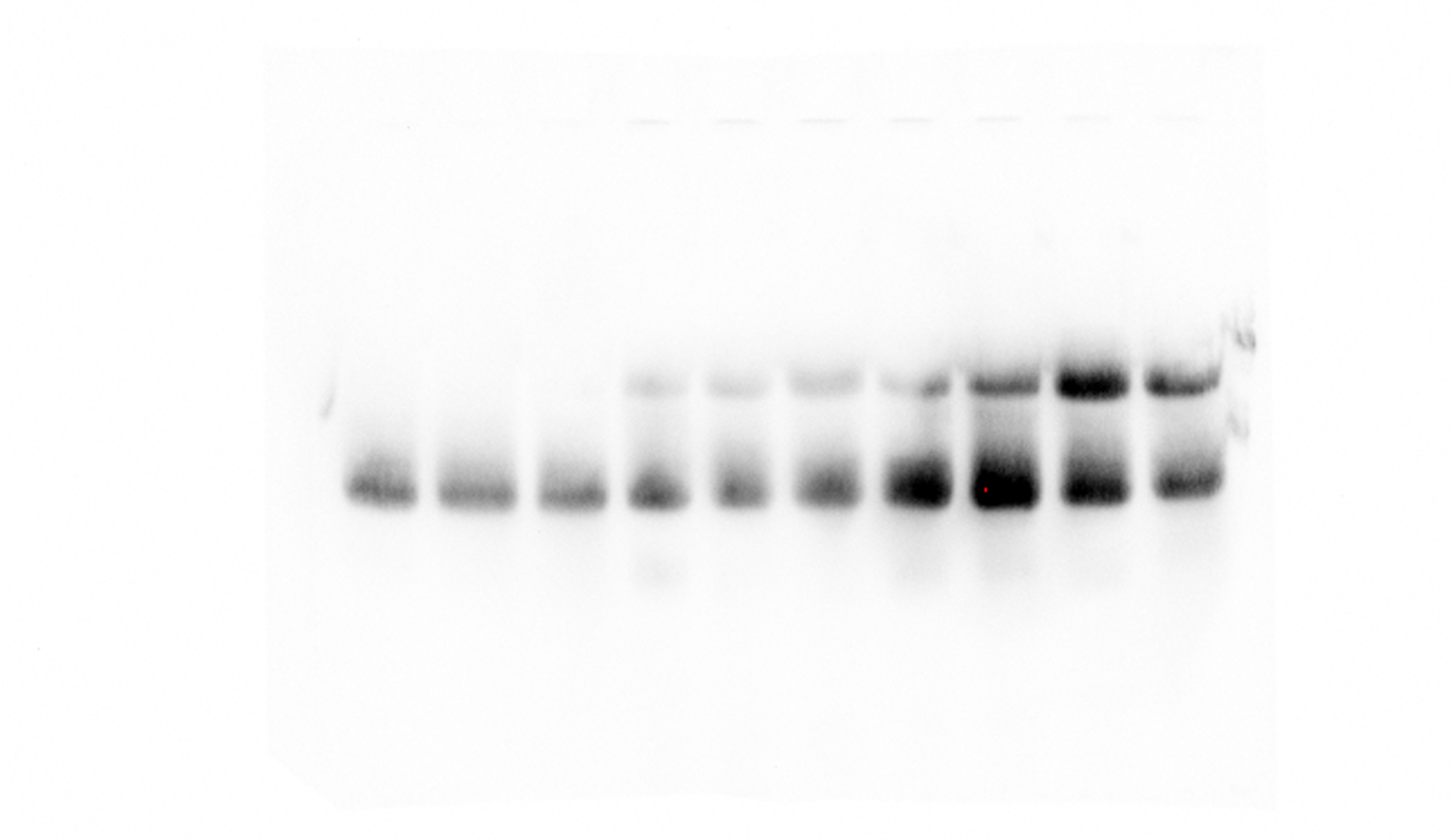

Supplement: Figure 6—source data 1. [file elife-96317-fig6-data1.zip › figure 6 source data 1/figure 6 source data 1.tif]

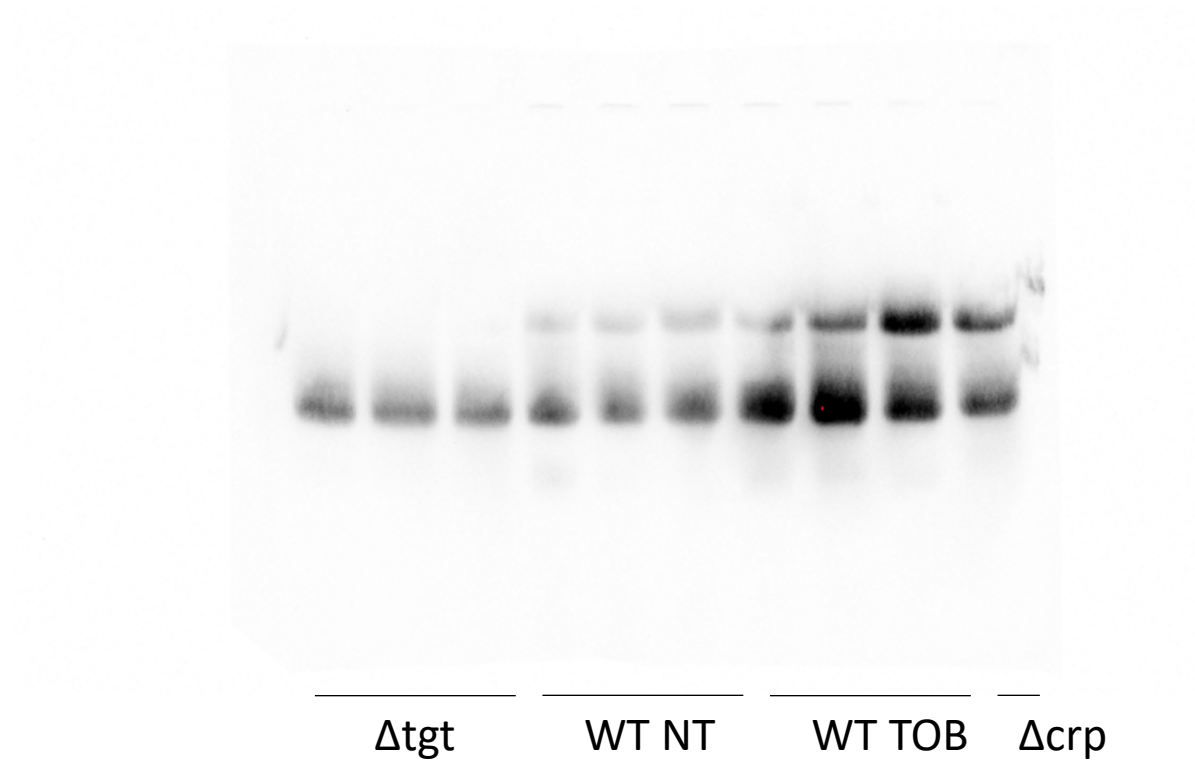

Supplement: Figure 6—source data 2. [file elife-96317-fig6-data2.zip › figure 6 source data 2/figure 6 source data 2.pdf]
