# Supplementary material for: Aminoglycoside tolerance in Vibrio cholerae engages translational reprogramming associated with queuosine tRNA modification
Source: eLife. 2025 Jan 6;13:RP96317. doi: 10.7554/eLife.96317 (PMC11703503; doi:10.7554/eLife.96317)
Supplement: Supplementary file 1. [file elife-96317-supp1.docx]

**Supplementary File 1: Proteomics identify differentially abundant proteins**

| Uniprot | Gene Name | Fasta headers | | | | | | | | | | | | |  |  |  |  |
| --- | --- | --- | --- | --- | --- | --- | --- | --- | --- | --- | --- | --- | --- | --- | --- | --- | --- | --- |
| I - More abundant in WT | | |  | | |  | | | |  | | | | |  |  |  |  |
| I – 1. PresentF606WT_AbsentJ420tgt | | |  | | | |  | | | |  | | | | |  |  |  |
| Q9KTY9 | tgt | Queuine tRNA-ribosyltransferase |  | | | |  | | | |  | | | | |  |  |  |
| Q9KNM6 | VC_2706 | queuosine precursor transporter |  | | | |  | | | |  | | | | |  |  |  |
| Q9KNH8 | VC_2761 | Bcr/CflA family efflux transporter |  | | | |  | | | |  | | | | |  |  |  |
| Q9KMV7 | VC_A0211 | Sensory box sensor histidine kinase |  | | | |  | | | |  | | | | |  |  |  |
| Q9KN55 | VC_A0110 | Uncharacterized protein |  | | | |  | | | |  | | | | |  |  |  |
| Q9KP41 | VC_2538 | Thiamine ABC transporter, permease protein, |  | | | |  | | | |  | | | | |  |  |  |
| Q9KTU0 | VC_0798 | Citrate lyase, beta subunit |  | | | |  | | | |  | | | | |  |  |  |
| Q9KSB8 | VC_1340 | PrpE protein |  | | | |  | | | |  | | | | |  |  |  |
| Q9KRW0 | VC_1524 | ABC transporter, permease protein |  | | | |  | | | |  | | | | |  |  |  |
| Q9KMB6 | VC_A0457 | Uncharacterized protein |  | | | |  | | | |  | | | | |  |  |  |
| Q9KUU0 | VC_0425 | lacZ |  | | | |  | | | |  | | | | |  |  |  |
| Q9KND5 | VC_A0030 | Uncharacterized protein |  | | | |  | | | |  | | | | |  |  |  |
| Q9KNP5 | zapB | Cell division protein ZapB |  | | | |  | | | |  | | | | |  |  |  |
| Q9KUS6 | VC_0439 | ThrE_2 domain-containing protein |  | | | |  | | | |  | | | | |  |  |  |
| Q9KLK9 | VC_A0734 | Uncharacterized protein |  | | | |  | | | |  | | | | |  |  |  |
| Q9KRP8 | VC_1588 | Transcriptional regulator, LysR family |  | | | |  | | | |  | | | | |  |  |  |
| Q9KRB9 | VC_1723 | TVP38/TMEM64 family membrane protein |  | | | |  | | | |  | | | | |  |  |  |
| Q9KNT7 | argB | Acetylglutamate kinase |  | | | |  | | | |  | | | | |  |  |  |
| Q9KRX3 | VC_1510 | Uncharacterized protein |  | | | |  | | | |  | | | | |  |  |  |
| Q9KPM7 | VC_2340 | HD-GYP domain-containing protein |  | | | |  | | | |  | | | | |  |  |  |
| Q9KL36 | VC_A0913 | Hemin ABC transporter, periplasmic hemin-binding protein HutB |  | | | |  | | | |  | | | | |  |  |  |
| Q9KVY3 | VC_0005 | Putative membrane protein insertion efficiency factor |  | | | |  | | | |  | | | | |  |  |  |
| Q9KQV1 | VC_1897 | Hit family protein |  | | | |  | | | |  | | | | |  |  |  |
| Q9KMY2 | VC_A0184 | cspE Cold shock DNA-binding domain protein |  | | |  | | | |  | | | | |  |  |  |  |
| Q9KL45 | VC_A0903 | Uncharacterized protein glycertae kinase |  | | |  | | | |  | | | | |  |  |  |  |
| Q9KRM7 | VC_1609 | Uncharacterized protein ABC-2 type transport system permease | |  | | | |  | | | |  | | | | |  |  |
| Q9KSC1 | VC_1337 | Citrate synthase | |  | | | |  | | | |  | | | | |  |  |
| Q9KLD9 | VC_A0807 | ABC transporter, periplasmic substrate-binding protein | |  | | | |  | | | |  | | | | |  |  |
| Q9KT63 | VC_1042 | Long-chain fatty acid transport protein | |  | | | |  | | | |  | | | | |  |  |
| Q9KN12 | VC_A0154 | Uncharacterized protein Na+:H+ antiporter subunit E | |  | | | |  | | | |  | | | | |  |  |
| Q9KN05 | tnaA | Tryptophanase | |  | | | |  | | | |  | | | | |  |  |
| Q9KS94 | queE | 7-carboxy-7-deazaguanine synthase | |  | | | |  | | | |  | | | | |  |  |
| Q9KQI2 | tmk | Thymidylate kinase | |  | | | |  | | | |  | | | | |  |  |
| Q9KVP6 | ubiC | Probable chorismate pyruvate-lyase | |  | | | |  | | | |  | | | | |  |  |
| Q9KMP2 | VC_A0281 | Integrase | |  | | | |  | | | |  | | | | |  |  |
| Q9KUW3 | VC_0396 | Transcriptional regulator, LuxR family | |  | | | |  | | | |  | | | | |  |  |
| Q9KUK3 | VC_0515 | Uncharacterized protein | |  | | | |  | | | |  | | | | |  |  |
| Q9KSC8 | VC_1330 | Uncharacterized protein | |  | | | |  | | | |  | | | | |  |  |
| Q9KUQ7 | VC_0458 | UPF0235 protein yggU | |  | | | |  | | | |  | | | | |  |  |
| I – 2. MoreF606wt_ThanJ420tgt | | | **log2FC WT/tgt** | | | **p value** | | | | **Adjusted p value** | | | | |  |  |  |  |
| Q9KTJ4 | gmhB | D-glycero-beta-D-manno-heptose-1,7-bisphosphate 7-phosphatase | 3,51 | | | 0,0001 | | | | 0,0005 | | | | |  |  |  |  |
| Q9KTU3 | VC_0795 | Citrate/sodium symporter | 3,46 | | | 0,0085 | | | | 0,0068 | | | | |  |  |  |  |
| Q9KSB4 | VC_1345 | Putative dioxygenase | 3,35 | | | 0,0024 | | | | 0,0034 | | | | |  |  |  |  |
| Q9KS61 | cheR2 | Chemotaxis protein methyltransferase | 2,93 | | | 0,0053 | | | | 0,0050 | | | | |  |  |  |  |
| Q9KN74 | VC_A0091 | UPF0251 protein VC_A0091 | 2,86 | | | 0,0013 | | | | 0,0025 | | | | |  |  |  |  |
| Q9KR81 | VC_1762 | EH_Signature domain-containing protein | 2,54 | | | 0,0023 | | | | 0,0034 | | | | |  |  |  |  |
| Q9KM90 | VC_A0491 | Uncharacterized protein | 2,26 | | | 0,0013 | | | | 0,0025 | | | | |  |  |  |  |
| Q9KQ10 | VC_2197 | Flagellar hook protein FlgE | 2,23 | | | 0,0036 | | | | 0,0042 | | | | |  |  |  |  |
| Q9KVI7 | murI | Glutamate racemase | 2,11 | | | 0,0015 | | | | 0,0028 | | | | |  |  |  |  |
| Q9KV46 | VC_0312 | NAD(P)H-flavin reductase | 2,10 | | | 0,0004 | | | | 0,0014 | | | | |  |  |  |  |
| Q9KL60 | VC_A0888 | Transcriptional regulator malT , LuxR family | 2,01 | | | 0,0038 | | | | 0,0042 | | | | |  |  |  |  |
| Q9KSV5 | VC_1151 | Uncharacterized protein lysO | 1,85 | | | 0,0003 | | | | 0,0012 | | | | |  |  |  |  |
| Q9KUR5 | VC_0450 | Membrane-bound lytic murein transglycosylase C | 1,81 | | | 0,0057 | | | | 0,0052 | | | | |  |  |  |  |
| Q9KVM8 | VC_0113 | ubiG? Methyltransferase-related protein | 1,76 | | | 0,0047 | | | | 0,0046 | | | | |  |  |  |  |
| Q9KPC7 | VC_2445 | gspA General secretion pathway protein A | 1,75 | | | 0,0039 | | | | 0,0042 | | | | |  |  |  |  |
| Q9KNL2 | nfuA | Fe/S biogenesis protein NfuA | 1,68 | | | 0,0010 | | | | 0,0022 | | | | |  |  |  |  |
| Q9KVI5 | VC_0161 | Transcriptional activator IlvY | 1,63 | | | 0,0128 | | | | 0,0090 | | | | |  |  |  |  |
| Q9KL10 | VC_A0940 | Transcriptional regulator, DeoR family | 1,62 | | | 0,0003 | | | | 0,0012 | | | | |  |  |  |  |
| Q9KR73 | VC_1770 | Uncharacterized protein | 1,52 | | | 0,0112 | | | | 0,0082 | | | | |  |  |  |  |
| Q9KKR5 | VC_A1037 | Amino acid ABC transporter, ATP-binding protein | 1,43 | | | 0,0080 | | | | 0,0065 | | | | |  |  |  |  |
| H9L4R1 | VC_0412 | MshO Uncharacterized protein | 1,43 | | | 0,0094 | | | | 0,0071 | | | | |  |  |  |  |
| Q9KKN4 | VC_A1068 | LRP Transcriptional regulator, AsnC family | 1,43 | | | 0,0061 | | | | 0,0053 | | | | |  |  |  |  |
| P45784 | epsN | Type II secretion system protein N | 1,38 | | | 0,0022 | | | | 0,0034 | | | | |  |  |  |  |
| Q9KU51 | VC_0673 | Probable membrane transporter protein | 1,37 | | | 0,0076 | | | | 0,0063 | | | | |  |  |  |  |
| Q9KNR9 | VC_2662 | Uncharacterized protein | 1,24 | | | 0,0008 | | | | 0,0019 | | | | |  |  |  |  |
| Q9KRY1 | rsmF | Ribosomal RNA small subunit methyltransferase F | 1,18 | | | 0,0060 | | | | 0,0053 | | | | |  |  |  |  |
| Q9KLX1 | VC_A0620 | Thiosulfate sulfurtransferase SseA, putative | 1,10 | | | 0,0020 | | | | 0,0034 | | | | |  |  |  |  |
| Q9KV88 | VC_0268 | ygaJ Uncharacterized protein | 1,09 | | | 0,0070 | | | | 0,0060 | | | | |  |  |  |  |
| Q9KPI7 | VC_2380 | Cobalamin biosynthesis protein CbiB, putative | 1,00 | | | 0,0042 | | | | 0,0042 | | | | |  |  |  |  |
| I – 3. PresentF606TOB_AbsentJ420TOB | | | | |  | | | |  | | | | |  | | | | |
| P0C6D1 | irgB | Iron-regulated virulence regulatory protein IrgB | | |  | | | |  | | | |  | | | | |  |
| P0C6D6 | tcpN | TCP pilus virulence regulatory protein | | |  | | | |  | | | |  | | | | |  |
| Q56632 | vibA | Vibriobactin-specific 2,3-dihydro-2,3-dihydroxybenzoate dehydrogenase | | |  | | | |  | | | |  | | | | |  |
| Q9KKP3 | VC_A1059 | Putative pseudouridine methyltransferase | | |  | | | |  | | | |  | | | | |  |
| Q9KL40 | hutX | Intracellular heme transport protein HutX | | |  | | | |  | | | |  | | | | |  |
| Q9KLG0 | fabV2 | Enoyl-[acyl-carrier-protein] reductase [NADH] 2 | | |  | | | |  | | | |  | | | | |  |
| Q9KLJ6 | glpB | Anaerobic glycerol-3-phosphate dehydrogenase subunit B | | |  | | | |  | | | |  | | | | |  |
| Q9KLR1 | VC_A0681 | 33-cGAMP-specific phosphodiesterase 1 | | |  | | | |  | | | |  | | | | |  |
| Q9KMY6 | pepT | Peptidase T | |  | | |  | | | |  | | | | |  |  |  |
| Q9KNL4 | bioH | Pimeloyl-[acyl-carrier protein] methyl ester esterase | |  | | |  | | | |  | | | | |  |  |  |
| Q9KPE3 | coaE | Dephospho-CoA kinase | |  | | |  | | | |  | | | | |  |  |  |
| Q9KRQ1 | VC_1585 | Catalase | |  | | |  | | | |  | | | | |  |  |  |
| Q9KS61 | cheR2 | Chemotaxis protein methyltransferase 2 | |  | | |  | | | |  | | | | |  |  |  |
| Q9KSB4 | VC_1345 | Putative dioxygenase | |  | | |  | | | |  | | | | |  |  |  |
| Q9KSW7 | hisI | Histidine biosynthesis bifunctional protein HisIE | |  | | |  | | | |  | | | | |  |  |  |
| Q9KTY9 | tgt | Queuine tRNA-ribosyltransferase | |  | | |  | | | |  | | | | |  |  |  |
| Q9KU27 | VC_0702 | Inosine/xanthosine triphosphatase | |  | | |  | | | |  | | | | |  |  |  |
| H9L4T3 | VC_2212 | Uncharacterized protein | |  | | |  | | | |  | | | | |  |  |  |
| Q9K2M8 | VC_A0348 | Uncharacterized protein | |  | | |  | | | |  | | | | |  |  |  |
| Q9KKK0 | VC_A1105 | DNA-binding response regulator | |  | | |  | | | |  | | | | |  |  |  |
| Q9KKX8 | VC_A0972 | MFS domain-containing protein | |  | | |  | | | |  | | | | |  |  |  |
| Q9KL71 | VC_A0876 | D-serine deaminase activator | |  | | |  | | | |  | | | | |  |  |  |
| Q9KLB3 | VC_A0833 | Transcriptional regulator, LysR family | |  | | |  | | | |  | | | | |  |  |  |
| Q9KLG6 | VC_A0778 | AHS2 domain-containing protein | |  | | |  | | | |  | | | | |  |  |  |
| Q9KLK5 | VC_A0738 | Uncharacterized protein | |  | | |  | | | |  | | | | |  |  |  |
| Q9KLK9 | VC_A0734 | Uncharacterized protein | |  | | |  | | | |  | | | | |  |  |  |
| Q9KLQ1 | VC_A0691 | Acetoacetyl-CoA reductase | |  | | |  | | | |  | | | | |  |  |  |
| Q9KM02 | VC_A0587 | PPC domain-containing protein | |  | | |  | | | |  | | | | |  |  |  |
| Q9KM77 | VC_A0511 | Anaerobic ribonucleoside-triphosphate reductase | |  | | |  | | | |  | | | | |  |  |  |
| Q9KM86 | VC_A0496 | Glutathione S-transferase, putative | |  | | |  | | | |  | | | | |  |  |  |
| Q9KM98 | VC_A0483 | Uncharacterized protein | |  | | |  | | | |  | | | | |  |  |  |
| Q9KMX3 | VC_A0193 | Na+/H+ antiporter, putative | |  | | |  | | | |  | | | | |  |  |  |
| Q9KN01 | VC_A0165 | GGDEF family protein | |  | | |  | | | |  | | | | |  |  |  |
| Q9KN09 | VC_A0157 | NADH dehydrogenase, putative | |  | | |  | | | |  | | | | |  |  |  |
| Q9KN25 | VC_A0141 | C4-dicarboxylate transport sensor protein, putative | |  | | |  | | | |  | | | | |  |  |  |
| Q9KN46 | VC_A0119 | ImpA_N domain-containing protein | |  | | |  | | | |  | | | | |  |  |  |
| Q9KN85 | VC_A0080 | GGDEF family protein | |  | | |  | | | |  | | | | |  |  |  |
| Q9KN89 | VC_A0076 | Gate domain-containing protein | |  | | |  | | | |  | | | | |  |  |  |
| Q9KN99 | VC_A0066 | Uncharacterized protein | |  | | |  | | | |  | | | | |  |  |  |
| Q9KNA1 | VC_A0064 | TonB system receptor, putative | |  | | |  | | | |  | | | | |  |  |  |
| Q9KNA2 | VC_A0063 | Protease II | |  | | |  | | | |  | | | | |  |  |  |
| Q9KNB4 | VC_A0051 | Uncharacterized protein | |  | | |  | | | |  | | | | |  |  |  |
| Q9KNE3 | VC_A0022 | Glutathione S-transfersae-related protein | |  | | |  | | | |  | | | | |  |  |  |
| Q9KNF6 | VC_A0008 | Methyl-accepting chemotaxis protein | |  | | |  | | | |  | | | | |  |  |  |
| Q9KNM6 | VC_2706 | Probable queuosine precursor transporter | |  | | |  | | | |  | | | | |  |  |  |
| Q9KNN0 | VC_2702 | Transcriptional regulator, LuxR family | |  | | |  | | | |  | | | | |  |  |  |
| Q9KPD7 | cpdA | 3,5-cyclic adenosine monophosphate phosphodiesterase CpdA | |  | | |  | | | |  | | | | |  |  |  |
| Q9KPJ7 | VC_2370 | Sensory box/GGDEF family protein | |  | | |  | | | |  | | | | |  |  |  |
| Q9KPR2 | VC_2304 | Uncharacterized protein | |  | | |  | | | |  | | | | |  |  |  |
| Q9KPY8 | VC_2224 | GGDEF family protein | |  | | |  | | | |  | | | | |  |  |  |
| Q9KQ04 | VC_2203 | Flagellar protein, putative | |  | | |  | | | |  | | | | |  |  |  |
| Q9KQ78 | fliP | Flagellar biosynthetic protein FliP | |  | | |  | | | |  | | | | |  |  |  |
| Q9KQN1 | VC_1967 | Methyl-accepting chemotaxis protein | |  | | |  | | | |  | | | | |  |  |  |
| Q9KQQ9 | VC_1939 | Uncharacterized protein | |  | | |  | | | |  | | | | |  |  |  |
| Q9KQW7 | VC_1880 | DUF2062 domain-containing protein | |  | | |  | | | |  | | | | |  |  |  |
| Q9KR48 | VC_1798 | Eha protein | |  | | |  | | | |  | | | | |  |  |  |
| Q9KRH6 | VC_1666 | VIBCH ABC transporter, ATP-binding protein, putative | |  | | |  | | | |  | | | | |  |  |  |
| Q9KRJ6 | VC_1644 | Uncharacterized protein | |  | | |  | | | |  | | | | |  |  |  |
| Q9KRL9 | VC_1617 | Transcriptional regulator, LysR family | |  | | |  | | | |  | | | | |  |  |  |
| Q9KRN2 | VC_1604 | Transcriptional regulatory protein | |  | | |  | | | |  | | | | |  |  |  |
| Q9KRW9 | VC_1515 | Chaperone, formate dehydrogenase-specific, putative | |  | | |  | | | |  | | | | |  |  |  |
| Q9KSC1 | VC_1337 | Citrate synthase | |  | | |  | | | |  | | | | |  |  |  |
| Q9KSC6 | VC_1332 | Uncharacterized protein | |  | | |  | | | |  | | | | |  |  |  |
| Q9KSC9 | VC_1329 | Opacity protein-related protein | |  | | |  | | | |  | | | | |  |  |  |
| Q9KSE2 | VC_1315 | Sensor histidine kinase | |  | | |  | | | |  | | | | |  |  |  |
| Q9KSP0 | VC_1216 | GGDEF family protein | |  | | |  | | | |  | | | | |  |  |  |
| Q9KSV5 | VC_1151 | Uncharacterized protein | |  | | |  | | | |  | | | | |  |  |  |
| Q9KT20 | VC_1085 | Sensor histidine kinase | |  | | |  | | | |  | | | | |  |  |  |
| Q9KT74 | VC_1031 | Inosine monophosphate dehydrogenase-related protein | |  | | |  | | | |  | | | | |  |  |  |
| Q9KTC3 | VC_0979 | Oxidoreductase, short-chain dehydrogenase/reductase family | |  | | |  | | | |  | | | | |  |  |  |
| Q9KTI4 | VC_0918 | UDP-N-acetyl-D-mannosaminuronic acid dehydrogenase | |  | | |  | | | |  | | | | |  |  |  |
| Q9KTV0 | VC_0787 | Transcriptional regulator, LysR family | |  | | |  | | | |  | | | | |  |  |  |
| Q9KU51 | VC_0673 | Probable membrane transporter protein | |  | | |  | | | |  | | | | |  |  |  |
| Q9KUQ1 | VC_0464 | Transcriptional regulator, LuxR family | |  | | |  | | | |  | | | | |  |  |  |
| Q9KUW6 | VC_0393 | Uncharacterized protein | |  | | |  | | | |  | | | | |  |  |  |
| Q9KV54 | VC_0303 | Sensor histidine kinase | |  | | |  | | | |  | | | | |  |  |  |
| Q9KVM2 | VC_0119 | Uroporphyrinogen-III synthase | |  | | |  | | | |  | | | | |  |  |  |
| Q9KVM8 | VC_0113 | Methyltransferase-related protein | |  | | |  | | | |  | | | | |  |  |  |
| Q9KVP4 | VC_0097 | Flagellar protein FliL | |  | | |  | | | |  | | | | |  |  |  |
| Q9KVS6 | VC_0063 | ThiF protein | |  | | |  | | | |  | | | | |  |  |  |
| I – 4. MoreF606TOB_ThanJ420TOB | | | **log2 FC WT/tgt** | | | **p value** | | | | **Adjusted p value** | | | | |  |  |  |  |
|  |  |  |  |  |  |  |  |  |  |  |  |  |  |  |  |  |  |  |
| Q9KMV8 | VC_A0210 | 33-cGAMP-specific phosphodiesterase 2 | 5,21 | | | 0,00000 | | | | 0,00005 | | | | |  |  |  |  |
| Q9KSR2 | VC_1194 | J domain-containing protein | 3,68 | | | 0,00929 | | | | 0,00488 | | | | |  |  |  |  |
| Q9KKY6 | VC_A0964 | Glycine cleavage operon activator, putative | 5,30 | | | 0,00002 | | | | 0,00043 | | | | |  |  |  |  |
| Q9KKL6 | VC_A1087 | Anti-sigma F factor antagonist, putative | 3,72 | | | 0,00060 | | | | 0,00202 | | | | |  |  |  |  |
| Q9KSK7 | VC_1249 | ACT domain-containing protein | 2,37 | | | 0,00973 | | | | 0,00488 | | | | |  |  |  |  |
| Q9KS89 | VC_1370 | GGDEF family protein | 3,51 | | | 0,00007 | | | | 0,00062 | | | | |  |  |  |  |
| Q9KNA0 | VC_A0065 | P/Homo B domain-containing protein | 3,48 | | | 0,00876 | | | | 0,00488 | | | | |  |  |  |  |
| Q9KT21 | VC_1084 | Sensory box sensor histidine kinase | 1,77 | | | 0,00183 | | | | 0,00235 | | | | |  |  |  |  |
| Q9KRA1 | VC_1741 | Transcriptional regulator, TetR family | 1,71 | | | 0,01423 | | | | 0,00606 | | | | |  |  |  |  |
| Q9KU00 | cutC | Copper homeostasis protein CutC | 1,85 | | | 0,01810 | | | | 0,00696 | | | | |  |  |  |  |
| Q9KTH7 | VC_0925 | Polysaccharide biosynthesis protein, putative | 3,09 | | | 0,00236 | | | | 0,00256 | | | | |  |  |  |  |
| Q9KSD3 | VC_1325 | Galactoside ABC transporter, periplasmic D-galactose/D-glucose-binding protein SV=1" | 1,77 | | | 0,02018 | | | | 0,00745 | | | | |  |  |  |  |
| Q9KUU2 | arcA | ARCA_VIBCH Arginine deiminase | 2,78 | | | 0,00191 | | | | 0,00235 | | | | |  |  |  |  |
| Q9KLB8 | phhA | PH4H_VIBCH Phenylalanine-4-hydroxylase | 2,16 | | | 0,03098 | | | | 0,00967 | | | | |  |  |  |  |
| Q9KPI7 | VC_2380 | Cobalamin biosynthesis protein CbiB, putative | 1,83 | | | 0,00973 | | | | 0,00488 | | | | |  |  |  |  |
| Q9KSE3 | VC_1314 | Transporter, putative | 2,64 | | | 0,02472 | | | | 0,00834 | | | | |  |  |  |  |
| Q9KQX3 | VC_1874 | Uncharacterized protein | 2,66 | | | 0,00403 | | | | 0,00309 | | | | |  |  |  |  |
| Q9KMJ2 | VC_A0356 | Uncharacterized protein | 2,60 | | | 0,00439 | | | | 0,00321 | | | | |  |  |  |  |
| Q9KRW3 | VC_1521 | Sensor histidine kinase | 2,69 | | | 0,00011 | | | | 0,00085 | | | | |  |  |  |  |
| Q9KN34 | rbsK | Ribokinase | 2,49 | | | 0,00270 | | | | 0,00269 | | | | |  |  |  |  |
| Q9KQX5 | VC_1872 | AAA_PrkA domain-containing protein | 2,94 | | | 0,00216 | | | | 0,00245 | | | | |  |  |  |  |
| Q9KS12 | rtxA | Multifunctional-autoprocessing repeats-in-toxin | 3,68 | | | 0,01920 | | | | 0,00716 | | | | |  |  |  |  |
| Q9KQ01 | VC_2206 | Uncharacterized protein | 2,76 | | | 0,00003 | | | | 0,00043 | | | | |  |  |  |  |
| P45774 | epsH | Type II secretion system protein H | 2,58 | | | 0,02202 | | | | 0,00772 | | | | |  |  |  |  |
| Q9KU02 | VC_0728 | PPK2 domain-containing protein | 3,08 | | | 0,01270 | | | | 0,00574 | | | | |  |  |  |  |
| Q9KKL7 | VC_A1086 | Response regulator | 2,65 | | | 0,02350 | | | | 0,00806 | | | | |  |  |  |  |
| Q9KSG5 | VC_1291 | Uncharacterized protein | 3,38 | | | 0,00096 | | | | 0,00223 | | | | |  |  |  |  |
| Q9KSV0 | VC_1156 | Sensor histidine kinase | 2,48 | | | 0,00113 | | | | 0,00224 | | | | |  |  |  |  |
| Q9KN48 | VC_A0117 | Sigma-54 dependent transcriptional regulator | 1,56 | | | 0,00974 | | | | 0,00488 | | | | |  |  |  |  |
| Q9KRG0 | VC_1682 | Peptide ABC transporter, permease protein | 2,56 | | | 0,00020 | | | | 0,00109 | | | | |  |  |  |  |
| Q9KRG1 | VC_1681 | Peptide ABC transporter, permease protein | 2,86 | | | 0,00015 | | | | 0,00099 | | | | |  |  |  |  |
| Q9KSD1 | mglA | Galactose/methyl galactoside import ATP-binding protein MglA | 2,08 | | | 0,02068 | | | | 0,00754 | | | | |  |  |  |  |
| Q9KLT8 | VC_A0653 | PTS system, sucrose-specific IIBC component | 2,43 | | | 0,00003 | | | | 0,00043 | | | | |  |  |  |  |
| P0C6Q8 | dam | DNA adenine methylase | 2,26 | | | 0,00448 | | | | 0,00324 | | | | |  |  |  |  |
| P0C6D3 | vibB | Vibriobactin-specific isochorismatase | 2,32 | | | 0,00194 | | | | 0,00235 | | | | |  |  |  |  |
| Q9KLF1 | VC_A0795 | Resolvase, putative | 2,33 | | | 0,00078 | | | | 0,00215 | | | | |  |  |  |  |
| Q9KNN7 | VC_2694 | Superoxide dismutase | 1,34 | | | 0,00647 | | | | 0,00419 | | | | |  |  |  |  |
| Q9KNW3 | VC_2617 | Arginine N-succinyltransferase OS=Vibrio cholerae serotype | 1,27 | | | 0,02884 | | | | 0,00915 | | | | |  |  |  |  |
| Q9KUA1 | VC_0622 | Histidine kinase | 1,89 | | | 0,00950 | | | | 0,00488 | | | | |  |  |  |  |
| Q9KQX9 | VC_1868 | Methyl-accepting chemotaxis protein | 2,27 | | | 0,01417 | | | | 0,00606 | | | | |  |  |  |  |
| Q9KS63 | VC_1397 | Chemotaxis protein CheA | 2,42 | | | 0,02557 | | | | 0,00852 | | | | |  |  |  |  |
| Q9KR16 | VC_1831 | Sensor histidine kinase | 2,54 | | | 0,00188 | | | | 0,00235 | | | | |  |  |  |  |
| Q9KKR5 | VC_A1037 | Amino acid ABC transporter, ATP-binding protein | 1,56 | | | 0,02096 | | | | 0,00754 | | | | |  |  |  |  |
| Q9KPK6 | grcA | GRCA_VIBCH Autonomous glycyl radical cofactor | 2,66 | | | 0,00332 | | | | 0,00269 | | | | |  |  |  |  |
| Q9KQX4 | VC_1873 | UPF0229 protein VC_1873 | 2,70 | | | 0,02180 | | | | 0,00770 | | | | |  |  |  |  |
| Q9KTI9 | VC_0913 | HlyD_D23 domain-containing protein | 1,71 | | | 0,02570 | | | | 0,00852 | | | | |  |  |  |  |
| Q9KTC7 | VC_0975 | NfeD domain-containing protein | 2,07 | | | 0,00048 | | | | 0,00177 | | | | |  |  |  |  |
| Q9KU65 | VC_0658 | C-di-GMP phosphodiesterase A-related protein | 2,14 | | | 0,00930 | | | | 0,00488 | | | | |  |  |  |  |
| Q9KNB6 | VC_A0049 | GGDEF family protein | 2,45 | | | 0,00175 | | | | 0,00235 | | | | |  |  |  |  |
| Q9KNY0 | VC_2600 | Uncharacterized protein | 1,81 | | | 0,00123 | | | | 0,00224 | | | | |  |  |  |  |
| Q9KTF2 | mrdA | Peptidoglycan D,D-transpeptidase MrdA | 1,65 | | | 0,01002 | | | | 0,00488 | | | | |  |  |  |  |
| Q9KQY2 | VC_1865 | Uncharacterized protein | 1,78 | | | 0,00316 | | | | 0,00269 | | | | |  |  |  |  |
| Q9KL39 | VC_A0909 | Oxygen-independent coproporphyrinogen III oxidase, putative | 2,04 | | | 0,01389 | | | | 0,00602 | | | | |  |  |  |  |
| Q9KVF6 | VC_0190 | DNA helicase uvrD | 1,94 | | | 0,01686 | | | | 0,00663 | | | | |  |  |  |  |
| Q9KUM9 | VC_0486 | Transcriptional regulator, DeoR family | 1,92 | | | 0,03066 | | | | 0,00961 | | | | |  |  |  |  |
| Q9KRI2 | VC_1659 | Uncharacterized protein | 2,10 | | | 0,01469 | | | | 0,00612 | | | | |  |  |  |  |
| Q9KNJ0 | VC_2748 | Nitrogen regulation protein | 1,87 | | | 0,00126 | | | | 0,00224 | | | | |  |  |  |  |
| Q9KP22 | VC_2557 | Uncharacterized protein OS | 1,81 | | | 0,00291 | | | | 0,00269 | | | | |  |  |  |  |
| Q9KRV1 | VC_1533 | DTW domain-containing protein | 1,83 | | | 0,00205 | | | | 0,00235 | | | | |  |  |  |  |
| Q9KQC8 | VC_2072 | Peptidase, insulinase family | 1,58 | | | 0,00893 | | | | 0,00488 | | | | |  |  |  |  |
| Q9KUM3 | VC_0492 | Uncharacterized protein | 2,34 | | | 0,00243 | | | | 0,00259 | | | | |  |  |  |  |
| Q9KQI3 | VC_2015 | DNA polymerase III, delta prime subunit | 1,77 | | | 0,00063 | | | | 0,00202 | | | | |  |  |  |  |
| Q9KPT7 | crl | Sigma factor-binding protein Crl | 1,58 | | | 0,00088 | | | | 0,00219 | | | | |  |  |  |  |
| Q9KS69 | VC_1390 | Transcriptional regulator, LysR family | 1,89 | | | 0,00007 | | | | 0,00062 | | | | |  |  |  |  |
| Q9KSC3 | VC_1335 | Transcriptional regulator, GntR family | 1,55 | | | 0,00321 | | | | 0,00269 | | | | |  |  |  |  |
| Q9KRL1 | VC_1629 | Uncharacterized protein | 1,89 | | | 0,00322 | | | | 0,00269 | | | | |  |  |  |  |
| Q9KLS5 | VC_A0666 | L-serine dehydratase | 1,71 | | | 0,00079 | | | | 0,00215 | | | | |  |  |  |  |
| Q9KM52 | VC_A0537 | Uncharacterized protein | 1,27 | | | 0,01088 | | | | 0,00511 | | | | |  |  |  |  |
| Q9KRI6 | VC_1655 | Magnesium transporter MgtE | 1,72 | | | 0,00363 | | | | 0,00282 | | | | |  |  |  |  |
| Q9KTJ1 | VC_0911 | Trehalose-6-phosphate hydrolase | 1,70 | | | 0,00042 | | | | 0,00166 | | | | |  |  |  |  |
| Q9KPZ9 | VC_2208 | Uncharacterized protein | 1,79 | | | 0,01534 | | | | 0,00629 | | | | |  |  |  |  |
| Q9KM32 | VC_A0557 | GGDEF family protein | 1,37 | | | 0,02135 | | | | 0,00762 | | | | |  |  |  |  |
| Q9KSA6 | VC_1353 | GGDEF family protein | 1,52 | | | 0,00156 | | | | 0,00231 | | | | |  |  |  |  |
| Q9KUC2 | VC_0600 | AB hydrolase-1 domain-containing protein | 1,72 | | | 0,00123 | | | | 0,00224 | | | | |  |  |  |  |
| Q9KM15 | VC_A0574 | N-acetyltransferase domain-containing protein | 1,39 | | | 0,01767 | | | | 0,00683 | | | | |  |  |  |  |
| Q9KS83 | VC_1376 | GGDEF family protein | 1,51 | | | 0,00363 | | | | 0,00282 | | | | |  |  |  |  |
| Q9KPT2 | VC_2280 | Uncharacterized protein | 1,45 | | | 0,00471 | | | | 0,00331 | | | | |  |  |  |  |
| Q9KVN8 | VC_0103 | Uncharacterized protein | 1,55 | | | 0,00249 | | | | 0,00262 | | | | |  |  |  |  |
| Q9KT24 | VC_1081 | Response regulator | 1,65 | | | 0,02632 | | | | 0,00861 | | | | |  |  |  |  |
| Q9KRJ1 | VC_1649 | Trypsin, putative | 1,54 | | | 0,00092 | | | | 0,00220 | | | | |  |  |  |  |
| Q9KLP6 | VC_A0697 | Sensory box/GGDEF family protein | 1,63 | | | 0,00022 | | | | 0,00109 | | | | |  |  |  |  |
| Q9KQ23 | ispE | ISPE_VIBCH 4-diphosphocytidyl-2-C-methyl-D-erythritol kinase ispE | 1,57 | | | 0,00119 | | | | 0,00224 | | | | |  |  |  |  |
| H9L4T1 | VC_A0450 | Uncharacterized protein | 1,76 | | | 0,00290 | | | | 0,00269 | | | | |  |  |  |  |
| Q9KU16 | VC_0714 | Uncharacterized protein | 1,60 | | | 0,02409 | | | | 0,00820 | | | | |  |  |  |  |
| Q9KRS6 | katG | KATG_VIBCH Catalase-peroxidase katG | 1,39 | | | 0,01256 | | | | 0,00571 | | | | |  |  |  |  |
| P29485 | tcpP | Toxin coregulated pilus biosynthesis protein P tcpP | 1,56 | | | 0,00124 | | | | 0,00224 | | | | |  |  |  |  |
| Q9KU53 | rppH | RPPH_VIBCH RNA pyrophosphohydrolase rppH | 2,03 | | | 0,00467 | | | | 0,00331 | | | | |  |  |  |  |
| Q9KKX3 | VC_A0977 | ABC transporter, ATP-binding protein | 1,84 | | | 0,02092 | | | | 0,00754 | | | | |  |  |  |  |
| Q9KQL5 | VC_1983 | Peptidase, putative | 2,47 | | | 0,02205 | | | | 0,00772 | | | | |  |  |  |  |
| Q9KSG0 | VC_1296 | Phosphomethylpyrimidine kinase | 3,54 | | | 0,00005 | | | | 0,00054 | | | | |  |  |  |  |
| Q9KPJ4 | VC_2373 | Glutamate synthase, large subunit | 1,53 | | | 0,00120 | | | | 0,00224 | | | | |  |  |  |  |
| Q9KS96 | VC_1363 | Siroheme synthase component enzyme | 1,31 | | | 0,00930 | | | | 0,00488 | | | | |  |  |  |  |
| Q9KP89 | VC_2484 | Long-chain-fatty-acid--CoA ligase, putative | 1,53 | | | 0,00535 | | | | 0,00367 | | | | |  |  |  |  |
| Q9KSM2 | VC_1234 | Exodeoxyribonuclease I | 1,59 | | | 0,02087 | | | | 0,00754 | | | | |  |  |  |  |
| Q9KSX5 | VC_1131 | Na_H_antiporter domain-containing protein | 2,07 | | | 0,01089 | | | | 0,00511 | | | | |  |  |  |  |
| Q9KTJ2 | VC_0910 | PTS system, trehalose-specific IIBC component | 1,33 | | | 0,00205 | | | | 0,00235 | | | | |  |  |  |  |
| Q9KKZ7 | VC_A0953 | Peptidyl-prolyl cis-trans isomerase C | 1,30 | | | 0,00997 | | | | 0,00488 | | | | |  |  |  |  |
| Q9KUU7 | VC_0418 | dTTP/UTP pyrophosphatase | 1,24 | | | 0,00258 | | | | 0,00267 | | | | |  |  |  |  |
| Q9KV39 | birA | Bifunctional ligase/repressor BirA | 1,47 | | | 0,00649 | | | | 0,00419 | | | | |  |  |  |  |
| Q9KS28 | VC_1433 | Uncharacterized protein | 1,23 | | | 0,00965 | | | | 0,00488 | | | | |  |  |  |  |
| Q9KMM4 | VC_A0307 | HNHc domain-containing protein | 1,46 | | | 0,00110 | | | | 0,00224 | | | | |  |  |  |  |
| Q9KUJ8 | VC_0522 | Beta-ketoadipate enol-lactone hydrolase, putative | 1,37 | | | 0,00202 | | | | 0,00235 | | | | |  |  |  |  |
| Q9KVT7 | purE | N5-carboxyaminoimidazole ribonucleotide mutase | 1,45 | | | 0,00886 | | | | 0,00488 | | | | |  |  |  |  |
| Q9KRU5 | VC_1539 | Probable ketoamine kinase VC_1539 | 1,09 | | | 0,01619 | | | | 0,00652 | | | | |  |  |  |  |
| Q9KSU7 | serC | Phosphoserine aminotransferase | 1,41 | | | 0,00147 | | | | 0,00231 | | | | |  |  |  |  |
| Q9KQU2 | VC_1906 | Methyltranfer_dom domain-containing protein | 2,00 | | | 0,00065 | | | | 0,00202 | | | | |  |  |  |  |
| Q9KRW4 | VC_1520 | ABC transporter, ATP-binding protein | 1,17 | | | 0,00887 | | | | 0,00488 | | | | |  |  |  |  |
| Q9KU52 | VC_0672 | Phosphoenolpyruvate-protein phosphotransferase | 1,41 | | | 0,00145 | | | | 0,00231 | | | | |  |  |  |  |
| Q9KLI6 | VC_A0758 | Arginine ABC transporter, permease protein | 1,24 | | | 0,01152 | | | | 0,00534 | | | | |  |  |  |  |
| Q9KPQ9 | panE | 2-dehydropantoate 2-reductase | 1,32 | | | 0,02013 | | | | 0,00745 | | | | |  |  |  |  |
| Q9KNW7 | VC_2613 | Phosphoribulokinase | 1,22 | | | 0,00346 | | | | 0,00278 | | | | |  |  |  |  |
| Q9KT18 | VC_1087 | Response regulator | 1,12 | | | 0,00908 | | | | 0,00488 | | | | |  |  |  |  |
| Q9KPP6 | recB | RecBCD enzyme subunit RecB | 1,24 | | | 0,00085 | | | | 0,00218 | | | | |  |  |  |  |
| Q9KQK1 | VC_1997 | Uncharacterized protein | 1,16 | | | 0,00200 | | | | 0,00235 | | | | |  |  |  |  |
| Q9KUN3 | argP | ARGP_VIBCH HTH-type transcriptional regulator ArgP | 1,53 | | | 0,00885 | | | | 0,00488 | | | | |  |  |  |  |
| Q9KMW8 | VC_A0198 | Site-specific DNA-methyltransferase, putative | 1,15 | | | 0,00300 | | | | 0,00269 | | | | |  |  |  |  |
| Q9KVB8 | VC_0228 | Uncharacterized protein | 1,27 | | | 0,00223 | | | | 0,00248 | | | | |  |  |  |  |
| Q9KVE7 | VC_0199 | Hemolysin secretion ATP-binding protein, putative | 1,29 | | | 0,00999 | | | | 0,00488 | | | | |  |  |  |  |
| Q9KQM5 | menB | 1,4-dihydroxy-2-naphthoyl-CoA synthase menB | 1,40 | | | 0,00328 | | | | 0,00269 | | | | |  |  |  |  |
| Q9KR89 | VC_1753 | Paraquat-inducible protein A | 1,11 | | | 0,00739 | | | | 0,00467 | | | | |  |  |  |  |
| Q9KQJ7 | VC_2001 | Putative glucose-6-phosphate 1-epimerase | 1,57 | | | 0,01040 | | | | 0,00501 | | | | |  |  |  |  |
| P57070 | lolB | Outer-membrane lipoprotein LolB | 1,04 | | | 0,01285 | | | | 0,00577 | | | | |  |  |  |  |
| Q9KL63 | VC_A0884 | Uncharacterized protein | 1,15 | | | 0,02695 | | | | 0,00863 | | | | |  |  |  |  |
| H9L4P1 | VC_0259 | Lipopolysaccharide biosynthesis protein RfbV | 1,21 | | | 0,00560 | | | | 0,00377 | | | | |  |  |  |  |
| Q9KMU4 | VC_A0225 | Glutamine amidotransferase type-2 domain-containing protein | 1,14 | | | 0,00164 | | | | 0,00235 | | | | |  |  |  |  |
| Q9KKU5 | VC_A1005 | Transcriptional regulator, MarR family | 1,14 | | | 0,02484 | | | | 0,00834 | | | | |  |  |  |  |
| Q9KVK1 | VC_0142 | DUF4145 domain-containing protein | 1,20 | | | 0,01451 | | | | 0,00609 | | | | |  |  |  |  |
| Q9KVB5 | VC_0231 | Uncharacterized protein | 1,09 | | | 0,00932 | | | | 0,00488 | | | | |  |  |  |  |
| Q9KKS4 | VC_A1026 | Uncharacterized protein | 1,31 | | | 0,00883 | | | | 0,00488 | | | | |  |  |  |  |
| Q9KUU0 | VC_0425 | Uncharacterized protein | 1,54 | | | 0,01872 | | | | 0,00702 | | | | |  |  |  |  |
| Q9KTA5 | VC_0998 | Uncharacterized protein | 1,15 | | | 0,00530 | | | | 0,00367 | | | | |  |  |  |  |
| Q9KNC4 | gltS | Sodium/glutamate symporter | 1,21 | | | 0,02667 | | | | 0,00862 | | | | |  |  |  |  |
| Q9KRS8 | VC_1558 | 6-phospho-beta-glucosidase | 1,37 | | | 0,00196 | | | | 0,00235 | | | | |  |  |  |  |
| Q9KMT9 | VC_A0230 | Iron(III) ABC transporter, ATP-binding protein | 1,08 | | | 0,01196 | | | | 0,00547 | | | | |  |  |  |  |
| Q9KPZ0 | VC_2222 | Smr domain-containing protein | 1,04 | | | 0,02562 | | | | 0,00852 | | | | |  |  |  |  |
| Q9KQE2 | VC_2058 | Uncharacterized protein | 1,05 | | | 0,00651 | | | | 0,00419 | | | | |  |  |  |  |
| P57066 | lolD | LOLD_VIBCH Lipoprotein-releasing system ATP-binding protein LolD | 1,01 | | | 0,00981 | | | | 0,00488 | | | | |  |  |  |  |
| Q9KLE5 | VC_A0801 | Q9KLE5_VIBCH Inosine-guanosine kinase | 1,00 | | | 0,02622 | | | | 0,00861 | | | | |  |  |  |  |
| Q9KS35 | VC_1426 | Spermidine/putrescine ABC transporter, permease protein | 1,11 | | | 0,00158 | | | | 0,00231 | | | | |  |  |  |  |
| Q9KSV4 | VC_1152 | HDOD domain-containing protein | 1,07 | | | 0,00926 | | | | 0,00488 | | | | |  |  |  |  |
| Q9KUM2 | VC_0493 | Q9KUM2_VIBCH Uncharacterized protein | 1,76 | | | 0,01375 | | | | 0,00600 | | | | |  |  |  |  |
| Q9KNA9 | VC_A0056 | Q9KNA9_VIBCH Transcriptional regulator, MerR family | 1,07 | | | 0,01454 | | | | 0,00609 | | | | |  |  |  |  |
| Q9KQ71 | VC_2130 | Flagellum-specific ATP synthase FliI | 1,15 | | | 0,00357 | | | | 0,00282 | | | | |  |  |  |  |
| Q9KLD8 | VC_A0808 | NodN-related protein | 1,27 | | | 0,00802 | | | | 0,00488 | | | | |  |  |  |  |
| Q9KP78 | VC_2497 | HD-GYP domain-containing protein | 1,09 | | | 0,00149 | | | | 0,00231 | | | | |  |  |  |  |
| Q60153 | tcpA | TCPA_VIBCH Toxin coregulated pilin | 1,01 | | | 0,02653 | | | | 0,00862 | | | | |  |  |  |  |
| Q9KRU1 | VC_1543 | Uncharacterized protein | 1,09 | | | 0,00268 | | | | 0,00269 | | | | |  |  |  |  |
| Q9KQ38 | VC_2166 | Trp repressor-binding protein | 1,46 | | | 0,01086 | | | | 0,00511 | | | | |  |  |  |  |
| Q9KQN3 | VC_1965 | TetR_C_33 domain-containing protein | 1,28 | | | 0,00698 | | | | 0,00445 | | | | |  |  |  |  |
| Q9KN86 | VC_A0079 | Uncharacterized protein | 1,23 | | | 0,00556 | | | | 0,00377 | | | | |  |  |  |  |
| Q9KRD3 | VC_1709 | Zinc protease, insulinase family | 1,25 | | | 0,00993 | | | | 0,00488 | | | | |  |  |  |  |
| Q9KKS7 | VC_A1023 | Uncharacterized protein | 1,16 | | | 0,00537 | | | | 0,00367 | | | | |  |  |  |  |
| Q9KVB7 | VC_0229 | Uncharacterized protein | 1,66 | | | 0,01838 | | | | 0,00696 | | | | |  |  |  |  |
| Q9KNR2 | VC_2669 | 5-carboxymethyl-2-hydroxymuconate delta isomerase, putative | 1,22 | | | 0,01524 | | | | 0,00628 | | | | |  |  |  |  |
| Q9KUI6 | mutS | MUTS_VIBCH DNA mismatch repair protein MutS | 1,07 | | | 0,00298 | | | | 0,00269 | | | | |  |  |  |  |
| Q9KVF0 | VC_0196 | ATP-dependent DNA helicase RecQ | 1,04 | | | 0,00303 | | | | 0,00269 | | | | |  |  |  |  |
| Q9KTJ3 | VC_0909 | Trehalose operon repressor | 1,03 | | | 0,00981 | | | | 0,00488 | | | | |  |  |  |  |
| Q9KVH1 | VC_0175 | Deoxycytidylate deaminase-related protein | 1,28 | | | 0,01307 | | | | 0,00584 | | | | |  |  |  |  |
| Q9KR77 | VC_1766 | Uncharacterized protein | 1,01 | | | 0,01408 | | | | 0,00606 | | | | |  |  |  |  |
| Q9KTL4 | truC | tRNA pseudouridine synthase C truC | 1,09 | | | 0,00416 | | | | 0,00310 | | | | |  |  |  |  |
| Q9KUM4 | VC_0491 | Uncharacterized protein | 1,10 | | | 0,00571 | | | | 0,00379 | | | | |  |  |  |  |
| P52022 | dnaE | DNA polymerase III subunit alpha | 1,07 | | | 0,00324 | | | | 0,00269 | | | | |  |  |  |  |
| Q9KP97 | VC_2476 | UPF0149 protein VC_2476 | 1,70 | | | 0,02715 | | | | 0,00865 | | | | |  |  |  |  |
| Q9KS92 | VC_1367 | GGDEF family protein | 1,19 | | | 0,00957 | | | | 0,00488 | | | | |  |  |  |  |
| Q9KU08 | ppx | Exopolyphosphatase ppx | 1,04 | | | 0,01694 | | | | 0,00663 | | | | |  |  |  |  |
| Q9KSI6 | VC_1270 | Glyoxylase II family protein | 1,32 | | | 0,01645 | | | | 0,00653 | | | | |  |  |  |  |
| Q9KRA6 | bpt | Aspartate/glutamate leucyltransferase bpt | 1,04 | | | 0,01838 | | | | 0,00696 | | | | |  |  |  |  |
| Q9KQF5 | topB | DNA topoisomerase 3 topB | 1,05 | | | 0,02938 | | | | 0,00928 | | | | |  |  |  |  |
| Q9KNF3 | malT | HTH-type transcriptional regulator MalT | 1,10 | | | 0,00227 | | | | 0,00249 | | | | |  |  |  |  |
| Q9KPE8 | VC_2420 | Flavodoxin | 1,39 | | | 0,00897 | | | | 0,00488 | | | | |  |  |  |  |
| Q9KNT4 | ppc | Phosphoenolpyruvate carboxylase ppc | 1,05 | | | 0,00302 | | | | 0,00269 | | | | |  |  |  |  |
| Q9KU29 | VC_0700 | Soluble lytic murein transglycosylase | 1,07 | | | 0,00291 | | | | 0,00269 | | | | |  |  |  |  |
| Q9KQV0 | VC_1898 | Methyl-accepting chemotaxis protein | 1,01 | | | 0,00574 | | | | 0,00379 | | | | |  |  |  |  |
| Q9KPE7 | VC_2421 | ampD protein | 1,04 | | | 0,02430 | | | | 0,00824 | | | | |  |  |  |  |
| Q9KU20 | rluD | Ribosomal large subunit pseudouridine synthase D rluD | 1,03 | | | 0,00290 | | | | 0,00269 | | | | |  |  |  |  |
| Q9KNQ5 | VC_2676 | Cell division protein FtsN, putative | 1,20 | | | 0,01452 | | | | 0,00609 | | | | |  |  |  |  |
| H9L4T5 | VC_0847 | Integrase, phage family | 1,28 | | | 0,02687 | | | | 0,00863 | | | | |  |  |  |  |
| Q9KQ28 | VC_2176 | UPF0162 protein VC_2176 | 1,04 | | | 0,00311 | | | | 0,00269 | | | | |  |  |  |  |
| Q9KUC0 | mrcB | PBPB_VIBCH Penicillin-binding protein 1B | 1,15 | | | 0,01050 | | | | 0,00502 | | | | |  |  |  |  |
| Q9KPV6 | uppS | UPPS_VIBCH Ditrans,polycis-undecaprenyl-diphosphate synthase | 1,03 | | | 0,00800 | | | | 0,00488 | | | | |  |  |  |  |
| Q9KVU5 | rsmB | RSMB_VIBCH Ribosomal RNA small subunit methyltransferase B | 1,02 | | | 0,01178 | | | | 0,00542 | | | | |  |  |  |  |
| Q9KMC3 | VC_A0441 | Uncharacterized protein | 1,17 | | | 0,00930 | | | | 0,00488 | | | | |  |  |  |  |
| Q9KVL9 | VC_0122 | Adenylate cyclase | 1,23 | | | 0,02356 | | | | 0,00806 | | | | |  |  |  |  |
| Q9KUW9 | metH | Methionine synthase | 1,28 | | | 0,02670 | | | | 0,00862 | | | | |  |  |  |  |
| Q9KL09 | VC_A0941 | Acyl-CoA thioester hydrolase-related protein | 1,03 | | | 0,02584 | | | | 0,00853 | | | | |  |  |  |  |
| Q9KMY4 | VC_A0182 | Sigma-54 dependent transcriptional regulator | 1,00 | | | 0,00859 | | | | 0,00488 | | | | |  |  |  |  |
| Q9KQ13 | flgH | Flagellar L-ring protein | 1,06 | | | 0,01335 | | | | 0,00593 | | | | |  |  |  |  |
| Q9KT38 | VC_1067 | GGDEF domain-containing protein | 1,04 | | | 0,02971 | | | | 0,00935 | | | | |  |  |  |  |
| Q9KM78 | VC_A0510 | Uncharacterized protein | 1,01 | | | 0,01640 | | | | 0,00653 | | | | |  |  |  |  |
| P0C6R0 | irgA | IRGA_VIBCH Iron-regulated outer membrane virulence protein | 1,03 | | | 0,02028 | | | | 0,00745 | | | | |  |  |  |  |
| Q9KPD8 | VC_2432 | Uncharacterized protein | 1,23 | | | 0,03115 | | | | 0,00968 | | | | |  |  |  |  |
| Q9KVS4 | thiG | THIG_VIBCH Thiazole synthase | 1,50 | | | 0,03191 | | | | 0,00983 | | | | |  |  |  |  |
| Q9KMG4 | higA-1 | Antitoxin HigA-1 | 1,30 | | | 0,01712 | | | | 0,00665 | | | | |  |  |  |  |
| Q9KUY5 | VC_0373 | Uncharacterized protein | 1,25 | | | 0,02357 | | | | 0,00806 | | | | |  |  |  |  |
| Q9KMG8 | VC_A0388 | Uncharacterized protein | 1,58 | | | 0,00081 | | | | 0,00215 | | | | |  |  |  |  |

| Uniprot | Gene Name | Fasta headers | | | | | | |  |
| --- | --- | --- | --- | --- | --- | --- | --- | --- | --- |
| II - More abundant in *∆tgt* | | | | | | | | |  |
| II – 1. PresentJ420tgt_AbsentF606wt | | | | | | | | |  |
| O34419 | rstR1 | Cryptic phage CTXphi transcriptional repressor RstR | |  | |  | |  | |
| Q9KN37 | rbsA | Ribose import ATP-binding protein RbsA | |  | |  | |  | |
| Q9KT77 | moaE | Molybdopterin synthase catalytic subunit | |  | |  | |  | |
| Q9KVG9 | vspR | Transcriptional regulator VspR | |  | |  | |  | |
| H9L4R3 | VC_A0444 | RelE protein | |  | |  | |  | |
| Q9KKW7 | VC_A0983 | L-lactate permease | |  | |  | |  | |
| Q9KL14 | VC_A0935 | Uncharacterized protein | |  | |  | |  | |
| Q9KLD8 | VC_A0808 | NodN-related protein | |  | |  | |  | |
| Q9KLF6 | VC_A0788 | DnaJ-related protein | |  | |  | |  | |
| Q9KLJ2 | VC_A0752 | Thioredoxin 2 | |  | |  | |  | |
| Q9KLV6 | VC_A0635 | Transcriptional regulator, LysR family | |  | |  | |  | |
| Q9KM02 | VC_A0587 | PPC domain-containing protein | |  | |  | |  | |
| Q9KM27 | VC_A0562 | Uncharacterized protein | |  | |  | |  | |
| Q9KMP9 | VC_A0271 | Uncharacterized protein | |  | |  | |  | |
| Q9KNC6 | VC_A0039 | Uncharacterized protein | |  | |  | |  | |
| Q9KNE3 | VC_A0022 | Glutathione S-transfersae-related protein | |  | |  | |  | |
| Q9KNI2 | VC_2757 | Uncharacterized protein | |  | |  | |  | |
| Q9KNM7 | VC_2705 | Sodium/solute symporter, putative | |  | |  | |  | |
| Q9KPF0 | VC_2418 | Thiol:disulfide interchange protein | |  | |  | |  | |
| Q9KQ76 | VC_2125 | Flagellar motor switch protein FliN | |  | |  | |  | |
| Q9KQG6 | VC_2032 | Uncharacterized protein | |  | |  | |  | |
| Q9KQS3 | VC_1925 | C4-dicarboxylate transport sensor protein | |  | |  | |  | |
| Q9KR71 | VC_1772 | WYL domain-containing protein | |  | |  | |  | |
| Q9KRG4 | VC_1678 | Phage shock protein A | |  | |  | |  | |
| Q9KRH0 | VC_1672 | DNA-3-methyladenine glycosidase I | |  | |  | |  | |
| Q9KRM1 | VC_1615 | Uncharacterized protein | |  | |  | |  | |
| Q9KRR5 | VC_1571 | Quinol oxidase, subunit I | |  | |  | |  | |
| Q9KSM4 | VC_1232 | Uncharacterized protein | |  | |  | |  | |
| Q9KSN9 | VC_1217 | N-acetyltransferase domain-containing protein | |  | |  | |  | |
| Q9KTS9 | VC_0809 | SWIM-type domain-containing protein | |  | |  | |  | |
| Q9KTV0 | VC_0787 | Transcriptional regulator, LysR family | |  | |  | |  | |
| Q9KUN2 | VC_0483 | Uncharacterized protein | |  | |  | |  | |
| Q9KUW6 | VC_0393 | Uncharacterized protein | |  | |  | |  | |
| Q9KVR9 | VC_0070 | Uncharacterized protein | |  | |  | |  | |
| II – 2. MoreJ420tgt_ThanF606wt | | | **log2 wt/tgt** | | **p value** | | **Adjusted p value** | |  |
| Q9KNX3 | kefG | Glutathione-regulated potassium-efflux system ancillary protein KefG | -7,30 | | 0,00001 | | 0,00036 | |  |
| Q9KLB8 | phhA | Phenylalanine-4-hydroxylase | -4,14 | | 0,00010 | | 0,00068 | |  |
| P09545 | hlyA | Hemolysin | -3,90 | | 0,00039 | | 0,00140 | |  |
| Q9KQN1 | VC_1967 | Methyl-accepting chemotaxis protein | -3,84 | | 0,00567 | | 0,00521 | |  |
| Q9KV16 | queG | Epoxyqueuosine reductase | -3,39 | | 0,00416 | | 0,00420 | |  |
| H9L4T3 | VC_2212 | Uncharacterized protein - putative Fe3+-citrate ABC transporter | -3,32 | | 0,00017 | | 0,00094 | |  |
| Q9KTJ9 | syd | Syd | -3,29 | | 0,00057 | | 0,00177 | |  |
| Q9KNW3 | VC_2617 | Arginine N-succinyltransferase | -2,93 | | 0,00002 | | 0,00050 | |  |
| Q9KU56 | mutH | DNA mismatch repair protein MutH | -2,90 | | 0,00004 | | 0,00050 | |  |
| Q9KTZ6 | VC_0734 | Malate synthase | -2,81 | | 0,00306 | | 0,00389 | |  |
| Q9KKQ7 | mtlA | PTS system mannitol-specific EIICBA component | -2,80 | | 0,00214 | | 0,00343 | |  |
| Q9KLB3 | VC_A0833 | Transcriptional regulator, LysR family | -2,66 | | 0,00379 | | 0,00420 | |  |
| Q9KTM2 | VC_0880 | Uncharacterized protein | -2,50 | | 0,00364 | | 0,00420 | |  |
| Q9KSQ4 | hutH | Histidine ammonia-lyase | -2,42 | | 0,00105 | | 0,00227 | |  |
| Q9KPR5 | VC_2301 | Transcriptional activator, putative | -2,39 | | 0,00075 | | 0,00195 | |  |
| Q9KNF6 | VC_A0008 | Methyl-accepting chemotaxis protein | -2,27 | | 0,00264 | | 0,00357 | |  |
| Q9KRF2 | VC_1690 | Alpha-1,6-galactosidase, putative | -2,25 | | 0,00022 | | 0,00110 | |  |
| Q9KS52 | VC_1408 | Transcriptional regulator, TetR family | -2,20 | | 0,00047 | | 0,00154 | |  |
| Q9KUN3 | argP | HTH-type transcriptional regulator ArgP | -2,20 | | 0,00006 | | 0,00050 | |  |
| Q9KU74 | VC_0649 | Transcriptional regulator, MarR family | -2,08 | | 0,00232 | | 0,00343 | |  |
| Q9KRA1 | VC_1741 | Transcriptional regulator, TetR family | -2,03 | | 0,00074 | | 0,00195 | |  |
| Q9KQ71 | VC_2130 | Flagellum-specific ATP synthase FliI | -1,89 | | 0,00016 | | 0,00094 | |  |
| Q9KUU2 | arcA | Arginine deiminase | -1,88 | | 0,00990 | | 0,00732 | |  |
| Q9KM69 | VC_A0519 | Fructose repressor | -1,80 | | 0,00183 | | 0,00331 | |  |
| Q9KS51 | VC_1409 | Multidrug resistance protein, putative | -1,77 | | 0,00895 | | 0,00699 | |  |
| Q9KS17 | VC_1444 | Uncharacterized protein | -1,73 | | 0,01228 | | 0,00882 | |  |
| P0C6Q5 | tcpF | Toxin coregulated pilus biosynthesis protein F | -1,72 | | 0,00006 | | 0,00050 | |  |
| Q9KVH8 | VC_0168 | Cytochrome c5 | -1,65 | | 0,00187 | | 0,00331 | |  |
| Q9KP69 | VC_2507 | PINc domain-containing protein | -1,62 | | 0,00903 | | 0,00699 | |  |
| Q9KM51 | VC_A0538 | Cytochrome b561, putative | -1,62 | | 0,00417 | | 0,00420 | |  |
| Q9KTY1 | VC_0749 | Iron-sulfur cluster assembly scaffold protein IscU | -1,59 | | 0,00072 | | 0,00195 | |  |
| Q9KMJ5 | VC_A0351 | Uncharacterized protein | -1,56 | | 0,00615 | | 0,00534 | |  |
| Q9KS13 | VC_1449 | Uncharacterized protein | -1,54 | | 0,01266 | | 0,00895 | |  |
| Q9KLM0 | VC_A0723 | 3-hydroxy-3-methylglutaryl CoA reductase | -1,53 | | 0,00266 | | 0,00357 | |  |
| Q9KV51 | VC_0306 | Thioredoxin | -1,50 | | 0,00255 | | 0,00357 | |  |
| Q9KRR1 | VC_1575 | Uncharacterized protein | -1,32 | | 0,00274 | | 0,00358 | |  |
| Q9F854 | hisD | Histidinol dehydrogenase | -1,28 | | 0,00385 | | 0,00420 | |  |
| Q9KUS1 | pdxA | 4-hydroxythreonine-4-phosphate dehydrogenase | -1,25 | | 0,00082 | | 0,00195 | |  |
| Q9KSI8 | VC_1268 | Uncharacterized protein | -1,13 | | 0,00714 | | 0,00600 | |  |
| Q9KPA8 | VC_2465 | Sigma-E factor regulatory protein RseB | -1,08 | | 0,00400 | | 0,00420 | |  |
| Q9KLG6 | VC_A0778 | AHS2 domain-containing protein | -1,08 | | 0,00343 | | 0,00420 | |  |
| Q9KVJ1 | VC_0153 | Uncharacterized protein | -1,05 | | 0,00936 | | 0,00708 | |  |
| II – 3. PresentJ420tgtTOB_AbsentF606wtTOB | | |  | |  | |  | |  |
| Q9KNM4 | gmk | Guanylate kinase | |  | |  | |  | |
| Q9KNC1 | VC_A0044 | Uncharacterized protein put mb protease | |  | |  | |  | |
| Q9KRS9 | VC_1557 | Transcriptional regulator, LacI family lipid A biosynthesis acyltransferase | |  | |  | |  | |
| Q9KQH2 | VC_2026 | Uncharacterized protein rRNA accumulation protein YceD | |  | |  | |  | |
| Q9KRR2 | VC_1574 | Uncharacterized protein put Transmembrane signal peptide protein | |  | |  | |  | |
| Q9KT04 | VC_1101 | Uncharacterized protein putative tryptophan/tyrosine transport system substrate-binding protein or T6SS | |  | |  | |  | |
| Q9KVH8 | VC_0168 | Cytochrome c5 | |  | |  | |  | |
| Q9KR31 | VC_1816 | Uncharacterized protein 1 HYP TRANSPORTER | |  | |  | |  | |
| Q9KQN0 | VC_1968 | Transcriptional regulator, HTH_3 family sutR regulator utilization of sulfur | |  | |  | |  | |
| Q9KNT7 | argB | Acetylglutamate kinase ornithine and arginine biosynthesis | |  | |  | |  | |
| Q9KU61 | VC_0662 | Branched-chain amino acid transport system carrier protein brnQ transport leucine, valine, and isoleucine | |  | |  | |  | |
| Q9KPR5 | VC_2301 | Transcriptional activator, putative putative anti-ECFsigma factor, ChrR | |  | |  | |  | |
| Q9KQ26 | prmC | Release factor glutamine methyltransferase | |  | |  | |  | |
| Q9KM00 | VC_A0589 | Peptide ABC transporter, permease protein, putative oligopeptide ABC transporter membrane subunit YejE | |  | |  | |  | |
| Q9KVR7 | VC_0072 | Sensory box/GGDEF family protein | |  | |  | |  | |
| II – 4. MoreJ420tgtTOB_ThanF606wtTOB | | | **log2 wt/tgt** | | **p value** | | **Adjusted p value** | |  |
| Q9KSP4 | VC_1212 | DNA polymerase | -2,38 | | 0,01099 | | 0,00512 | |  |
| Q9KR61 | nanK | N-acetylmannosamine kinase | -4,31 | | 0,00003 | | 0,00043 | |  |
| Q9KT86 | rnfA/rsxA | Ion-translocating oxidoreductase complex subunit A | -3,56 | | 0,00002 | | 0,00043 | |  |
| Q9KRT1 | VC_1555 | Uncharacterized protein | -1,48 | | 0,00902 | | 0,00488 | |  |
| Q9KV51 | VC_0306 | Thioredoxin trxA | -1,69 | | 0,02226 | | 0,00776 | |  |
| Q9KM51 | VC_A0538 | Cytochrome b561, putative | -2,87 | | 0,00756 | | 0,00474 | |  |
| Q9KNR9 | VC_2662 | Uncharacterized protein | -1,31 | | 0,03255 | | 0,00995 | |  |
| Q9KR73 | VC_1770 | Uncharacterized protein | -1,31 | | 0,01849 | | 0,00696 | |  |
| P0C6C8 | fur | Ferric uptake regulation protein | -2,39 | | 0,00030 | | 0,00141 | |  |
| Q9KMN5 | VC_A0293 | Uncharacterized protein | -2,14 | | 0,00017 | | 0,00099 | |  |
| Q9KV88 | VC_0268 | Uncharacterized protein | -2,11 | | 0,00406 | | 0,00309 | |  |
| Q9KNQ9 | rraA | Regulator of ribonuclease activity A | -2,42 | | 0,00310 | | 0,00269 | |  |
| Q9KPZ5 | VC_2217 | Beta-N-acetylhexosaminidase | -2,91 | | 0,00054 | | 0,00191 | |  |
| Q9KTN7 | tadA | tRNA-specific adenosine deaminase | -2,04 | | 0,00110 | | 0,00224 | |  |
| Q9KRP5 | VC_1591 | Oxidoreductase, short-chain dehydrogenase/reductase family | -2,14 | | 0,00804 | | 0,00488 | |  |
| P0C6C4 | flaB | Flagellin B | -1,88 | | 0,00131 | | 0,00228 | |  |
| Q9KTZ8 | VC_0732 | Transcriptional regulator, LysR family oxyR like | -1,96 | | 0,00070 | | 0,00210 | |  |
| Q9KSV6 | VC_1150 | Uncharacterized protein | -1,29 | | 0,01007 | | 0,00488 | |  |
| Q9KVM9 | VC_0112 | Cytochrome c4 | -2,60 | | 0,01486 | | 0,00616 | |  |
| Q9KVK6 | cdgJ | Cyclic di-GMP phosphodiesterase CdgJ | -3,89 | | 0,00017 | | 0,00099 | |  |
| Q9KR96 | VC_1746 | Transcriptional regulator, TetR family | -1,85 | | 0,00299 | | 0,00269 | |  |
| P0C6C5 | flaC | Flagellin C OS=Vibrio cholerae serotype | -1,82 | | 0,00042 | | 0,00166 | |  |
| Q9KPX7 | VC_2235 | Methyltransf_11 domain-containing protein | -1,53 | | 0,00144 | | 0,00231 | |  |
| Q9KL76 | VC_A0871 | Transcriptional regulator, GntR family | -1,20 | | 0,00284 | | 0,00269 | |  |
| Q9KQ58 | VC_2146 | Uncharacterized protein | -1,38 | | 0,00181 | | 0,00235 | |  |
| Q9KKP2 | ribB | 3,4-dihydroxy-2-butanone 4-phosphate synthase | -2,73 | | 0,00036 | | 0,00157 | |  |
| Q9KNL7 | greB | Transcription elongation factor GreB | -2,29 | | 0,02154 | | 0,00765 | |  |
| Q9KV61 | VC_0296 | Biotin carboxyl carrier protein of acetyl-CoA carboxylase | -1,70 | | 0,00429 | | 0,00317 | |  |
| Q9KUN2 | VC_0483 | Uncharacterized protein | -1,46 | | 0,00180 | | 0,00235 | |  |
| P0C6P9 | tpx | Thiol peroxidase | -1,42 | | 0,01847 | | 0,00696 | |  |
| Q9KN91 | VC_A0074 | GGDEF family protein | -1,35 | | 0,00934 | | 0,00488 | |  |
| Q9KV27 | nudC | NADH pyrophosphatase | -1,38 | | 0,00104 | | 0,00224 | |  |
| Q9KTF3 | mrdB | Peptidoglycan glycosyltransferase | -1,35 | | 0,00766 | | 0,00476 | |  |
| Q9F854 | hisD | Histidinol dehydrogenase | -1,60 | | 0,00994 | | 0,00488 | |  |
| Q9KV12 | miaA | tRNA dimethylallyltransferase | -1,44 | | 0,00177 | | 0,00235 | |  |
| Q9KT82 | VC_1023 | Putative gluconeogenesis factor | -1,72 | | 0,02103 | | 0,00754 | |  |
| Q9KKQ1 | VC_A1051 | Uncharacterized protein | -1,32 | | 0,00452 | | 0,00324 | |  |
| Q9KKZ9 | VC_A0951 | UPF0145 protein VC_A0951 | -1,30 | | 0,01376 | | 0,00600 | |  |
| Q9KLK6 | luxP | Autoinducer 2-binding periplasmic protein LuxP | -1,19 | | 0,00413 | | 0,00310 | |  |
| Q9KTX4 | ndk | Nucleoside diphosphate kinase | -1,10 | | 0,01640 | | 0,00653 | |  |
| Q9KMJ8 | VC_A0345 | Uncharacterized protein | -1,18 | | 0,01699 | | 0,00663 | |  |
| Q9KU82 | rimP | Ribosome maturation factor RimP | -1,24 | | 0,01367 | | 0,00600 | |  |
| Q9KSF3 | VC_1303 | Para-aminobenzoate synthase, component I | -1,14 | | 0,02352 | | 0,00806 | |  |
| Q9KS93 | queC | 7-cyano-7-deazaguanine synthase | -1,01 | | 0,01568 | | 0,00635 | |  |
| Q9KQM0 | VC_1978 | 5-deoxynucleotidase | -1,18 | | 0,00153 | | 0,00231 | |  |
| Q9KST2 | trpE | Anthranilate synthase component 1 | -1,86 | | 0,03127 | | 0,00968 | |  |
| Q9KN74 | VC_A0091 | UPF0251 protein | -1,10 | | 0,01555 | | 0,00634 | |  |
| Q9KL95 | VC_A0851 | HATPase_c domain-containing protein | -1,55 | | 0,00154 | | 0,00231 | |  |
| Q9KNJ9 | VC_2739 | AsmA domain-containing protein | -1,16 | | 0,03228 | | 0,00991 | |  |
