# Supplementary material for: Aminoglycoside tolerance in Vibrio cholerae engages translational reprogramming associated with queuosine tRNA modification
Source: eLife. 2025 Jan 6;13:RP96317. doi: 10.7554/eLife.96317 (PMC11703503; doi:10.7554/eLife.96317)
Supplement: Supplementary file 2. [file elife-96317-supp2.docx]

**Supplementary File 2. Ribosome profiling.**

| ID | log2FoldChange | p value adj |
| --- | --- | --- |
| Transcripts UP |  |  |
| *VC_RS08405* | 5,16 | 2,54E-15 |
| *VC_RS08700* | 4,78 | 5,99E-03 |
| *glpD* | 4,19 | 1,40E-25 |
| *VC_RS00110* | 3,89 | 4,75E-13 |
| *VC_RS08400* | 3,83 | 3,23E-47 |
| *VC_RS07070* | 3,78 | 2,77E-05 |
| *VC_RS16920* | 3,64 | 9,32E-14 |
| *iscR* | 3,56 | 1,67E-24 |
| *VC_RS15320* | 3,47 | 4,97E-03 |
| *VC_RS16810* | 3,26 | 4,95E-04 |
| *hscB* | 3,14 | 6,58E-29 |
| *fadE* | 3,01 | 4,79E-14 |
| *siaQ* | 2,98 | 5,85E-06 |
| *VC_RS12540* | 2,90 | 2,09E-07 |
| *VC_RS15715* | 2,87 | 3,51E-03 |
| *VC_RS03415* | 2,76 | 5,14E-06 |
| *pdhR* | 2,69 | 4,02E-17 |
| *fadB* | 2,69 | 3,71E-16 |
| *VC_RS05835* | 2,66 | 2,21E-11 |
| *VC_RS12625* | 2,63 | 9,15E-17 |
| *VC_RS15780* | 2,57 | 3,00E-05 |
| *VC_RS06745* | 2,56 | 1,00E-03 |
| *VC_RS02460* | 2,53 | 4,43E-11 |
| *VC_RS15675* | 2,51 | 1,77E-07 |
| *VC_RS03375* | 2,50 | 3,27E-13 |
| *VC_RS15660* | 2,50 | 7,01E-08 |
| *VC_RS12840* | 2,45 | 1,93E-07 |
| *astA* | 2,43 | 3,51E-08 |
| *VC_RS18335* | 2,40 | 4,25E-05 |
| *VC_RS18385* | 2,40 | 6,53E-07 |
| *VC_RS18760* | 2,38 | 5,01E-03 |
| *VC_RS15265* | 2,37 | 1,85E-06 |
| *VC_RS15655* | 2,35 | 1,32E-08 |
| *VC_RS09590* | 2,34 | 2,10E-16 |
| *VC_RS17980* | 2,30 | 2,40E-07 |
| *rluC* | 2,30 | 3,62E-12 |
| *VC_RS08995* | 2,28 | 3,72E-05 |
| *VC_RS15750* | 2,25 | 9,78E-13 |
| *VC_RS06650* | 2,22 | 2,84E-03 |
| *VC_RS06240* | 2,21 | 3,77E-07 |
| *VC_RS17900* | 2,20 | 9,26E-06 |
| *dinB* | 2,19 | 6,60E-05 |
| *VC_RS16075* | 2,18 | 1,66E-06 |
| *glpK* | 2,13 | 5,64E-06 |
| *VC_RS03935* | 2,13 | 9,02E-06 |
| *VC_RS03750* | 2,13 | 3,89E-15 |
| *VC_RS09530* | 2,11 | 1,28E-03 |
| *iscA* | 2,10 | 7,00E-11 |
| *VC_RS18910* | 2,02 | 1,35E-03 |
| *VC_RS18595* | 2,00 | 3,76E-06 |
| *VC_RS06645* | 1,99 | 5,86E-04 |
| *VC_RS17880* | 1,97 | 1,27E-06 |
| *VC_RS17250* | 1,96 | 1,02E-04 |
| *VC_RS14235* | 1,95 | 7,29E-04 |
| *vpsQ* | 1,94 | 1,63E-05 |
| *VC_RS15795* | 1,92 | 3,27E-05 |
| *pncB* | 1,92 | 2,09E-11 |
| *VC_RS14320* | 1,91 | 3,76E-03 |
| *VC_RS19075* | 1,89 | 1,12E-06 |
| *VC_RS18065* | 1,88 | 2,22E-05 |
| *VC_RS05480* | 1,86 | 1,03E-03 |
| *VC_RS09310* | 1,86 | 1,69E-03 |
| *VC_RS16025* | 1,83 | 1,60E-07 |
| *VC_RS05200* | 1,82 | 3,76E-03 |
| *VC_RS06880* | 1,81 | 4,25E-06 |
| *VC_RS03615* | 1,81 | 9,36E-03 |
| *sdhC* | 1,80 | 8,98E-06 |
| *VC_RS12765* | 1,80 | 4,08E-06 |
| *vceC* | 1,77 | 2,69E-06 |
| *VC_RS08690* | 1,77 | 1,16E-03 |
| *VC_RS18420* | 1,75 | 4,95E-06 |
| *VC_RS06145* | 1,75 | 9,56E-08 |
| *VC_RS13705* | 1,73 | 3,89E-03 |
| *VC_RS09775* | 1,72 | 1,29E-05 |
| *VC_RS00830* | 1,72 | 2,06E-09 |
| *VC_RS15990* | 1,71 | 4,18E-04 |
| *astD* | 1,69 | 4,99E-05 |
| *rmuC* | 1,69 | 3,91E-06 |
| *cobA* | 1,67 | 6,18E-03 |
| *VC_RS06730* | 1,67 | 9,56E-03 |
| *VC_RS03925* | 1,67 | 2,64E-06 |
| *rpoE* | 1,65 | 3,06E-04 |
| *VC_RS13840* | 1,64 | 1,26E-06 |
| *pspC* | 1,62 | 5,50E-04 |
| *VC_RS05485* | 1,61 | 7,43E-04 |
| *VC_RS13775* | 1,60 | 2,22E-04 |
| *VC_RS13830* | 1,59 | 1,63E-05 |
| *VC_RS05795* | 1,59 | 8,95E-07 |
| *VC_RS04070* | 1,57 | 1,90E-04 |
| *pspA* | 1,55 | 6,11E-04 |
| *ectB* | 1,54 | 6,29E-03 |
| *VC_RS18060* | 1,53 | 9,22E-06 |
| *bioA* | 1,53 | 4,28E-03 |
| *VC_RS17350* | 1,50 | 2,41E-05 |
| *VC_RS16055* | 1,49 | 3,45E-04 |
| *VC_RS03930* | 1,49 | 2,68E-05 |
| *hppD* | 1,49 | 1,68E-03 |
| *rmf* | 1,48 | 6,04E-03 |
| *VC_RS18045* | 1,48 | 3,59E-03 |
| *pgl* | 1,47 | 3,65E-04 |
| *VC_RS09410* | 1,46 | 1,81E-03 |
| *VC_RS15825* | 1,46 | 8,01E-04 |
| *VC_RS01860* | 1,46 | 4,25E-03 |
| *VC_RS00045* | 1,46 | 2,22E-04 |
| *VC_RS08415* | 1,45 | 5,19E-05 |
| *thiC* | 1,44 | 3,32E-05 |
| *VC_RS03545* | 1,43 | 5,16E-04 |
| *VC_RS07590* | 1,42 | 2,00E-03 |
| *bioF* | 1,39 | 3,28E-05 |
| *vcrM* | 1,39 | 1,90E-03 |
| *VC_RS11130* | 1,38 | 4,37E-03 |
| *VC_RS13575* | 1,38 | 3,80E-03 |
| *thiD* | 1,37 | 1,07E-05 |
| *VC_RS17170* | 1,37 | 5,86E-04 |
| *VC_RS00235* | 1,37 | 1,19E-04 |
| *VC_RS06940* | 1,37 | 1,53E-05 |
| *purM* | 1,37 | 5,79E-04 |
| *emrD* | 1,35 | 1,37E-04 |
| *rpoS* | 1,32 | 9,84E-06 |
| *rpsN* | 1,31 | 5,86E-04 |
| *iscU* | 1,31 | 3,91E-04 |
| *VC_RS05395* | 1,30 | 3,89E-03 |
| *purN* | 1,30 | 6,54E-05 |
| *VC_RS08990* | 1,29 | 2,00E-03 |
| *truB* | 1,28 | 4,26E-04 |
| *rnc* | 1,28 | 4,81E-03 |
| *VC_RS16325* | 1,28 | 3,14E-03 |
| *miaB* | 1,25 | 1,55E-06 |
| *VC_RS00525* | 1,24 | 1,94E-03 |
| *VC_RS00310* | 1,24 | 2,05E-03 |
| *recN* | 1,23 | 1,53E-04 |
| *VC_RS00450* | 1,23 | 6,52E-03 |
| *norR* | 1,22 | 7,84E-04 |
| *rpmG* | 1,22 | 9,32E-03 |
| *VC_RS05080* | 1,20 | 3,77E-06 |
| *VC_RS00360* | 1,20 | 7,07E-04 |
| *VC_RS17040* | 1,20 | 2,06E-03 |
| *VC_RS11880* | 1,20 | 5,73E-05 |
| *zwf* | 1,19 | 9,56E-05 |
| *rimO* | 1,18 | 8,37E-04 |
| *xseB* | 1,18 | 1,46E-03 |
| *fadA* | 1,17 | 5,74E-03 |
| *pspB* | 1,17 | 7,83E-03 |
| *nhaA* | 1,17 | 2,57E-03 |
| *gspI* | 1,16 | 3,74E-03 |
| *VC_RS00180* | 1,16 | 1,52E-03 |
| *rluB* | 1,16 | 8,17E-05 |
| *VC_RS05405* | 1,15 | 2,69E-03 |
| *queE* | 1,15 | 9,97E-03 |
| *erpA* | 1,12 | 9,42E-03 |
| *VC_RS05410* | 1,11 | 2,47E-03 |
| *dbpA* | 1,06 | 7,53E-03 |
| *VC_RS15770* | 1,06 | 2,50E-03 |
| *VC_RS05800* | 1,06 | 1,80E-03 |
| *VC_RS15765* | 1,05 | 4,86E-03 |
| *ilvG* | 1,02 | 9,03E-04 |
| *VC_RS17205* | 1,02 | 2,74E-03 |
| *cgtA* | 1,02 | 2,06E-04 |
| *VC_RS08280* | 1,01 | 7,16E-04 |
| *lepA* | 0,98 | 1,30E-03 |
| *secD* | 0,97 | 1,44E-04 |
| *secD* | 0,97 | 1,44E-04 |
| *thiI* | 0,95 | 9,17E-03 |
| *yihI* | 0,94 | 7,72E-03 |
| *nlpD* | 0,93 | 6,99E-03 |
| *lipB* | 0,93 | 3,85E-03 |
| *VC_RS03795* | 0,92 | 6,44E-03 |
| *rpoH* | 0,91 | 2,26E-04 |
| *VC_RS07895* | 0,86 | 9,32E-03 |
| *ruvA* | 0,86 | 4,66E-03 |
| *ruvB* | 0,83 | 7,43E-03 |
| *mgtE* | 0,83 | 2,99E-03 |
| *mgtE* | 0,83 | 2,99E-03 |
| *der* | 0,82 | 2,14E-03 |
| *lepB* | 0,77 | 9,17E-03 |
| Transcripts DOWN |  |  |
| *tgt* | -5,96 | 1,33E-07 |
| *treC* | -4,79 | 2,04E-86 |
| *VC_RS07220* | -4,54 | 2,03E-02 |
| *VC_RS06415* | -4,35 | 1,81E-24 |
| *VC_RS08165* | -4,34 | 5,68E-48 |
| *VC_RS06535* | -4,33 | 4,12E-20 |
| *treB* | -4,26 | 3,95E-77 |
| *dcuC* | -4,25 | 5,49E-40 |
| *frdD* | -4,02 | 3,04E-05 |
| *VC_RS13520* | -4,01 | 8,27E-03 |
| *VC_RS12800* | -4,01 | 2,09E-15 |
| *VC_RS06405* | -3,90 | 5,49E-40 |
| *VC_RS18125* | -3,89 | 1,70E-24 |
| *adhE* | -3,85 | 4,00E-38 |
| *VC_RS03955* | -3,85 | 1,12E-06 |
| *VC_RS03950* | -3,76 | 2,56E-13 |
| *frdC* | -3,75 | 2,75E-14 |
| *VC_RS03300* | -3,73 | 7,21E-21 |
| *VC_RS06410* | -3,66 | 1,68E-19 |
| *VC_RS09000* | -3,62 | 6,89E-35 |
| *frdA* | -3,50 | 3,01E-12 |
| *VC_RS16590* | -3,49 | 2,26E-11 |
| *VC_RS18120* | -3,45 | 5,19E-20 |
| *VC_RS11425* | -3,44 | 3,82E-18 |
| *VC_RS17100* | -3,42 | 5,40E-24 |
| *grcA* | -3,39 | 4,67E-14 |
| *nrdD* | -3,38 | 6,19E-33 |
| *VC_RS14495* | -3,36 | 4,85E-02 |
| *pepT* | -3,35 | 8,01E-18 |
| *menC* | -3,34 | 2,34E-16 |
| *VC_RS03305* | -3,30 | 5,57E-23 |
| *VC_RS13030* | -3,30 | 2,56E-03 |
| *VC_RS09030* | -3,16 | 1,21E-22 |
| *VC_RS10340* | -3,13 | 1,28E-10 |
| *VC_RS09395* | -3,12 | 4,11E-18 |
| *VC_RS18865* | -3,06 | 6,78E-05 |
| *VC_RS09390* | -3,06 | 2,71E-20 |
| *menE* | -3,05 | 1,43E-13 |
| *yccS* | -2,99 | 1,50E-20 |
| *pykF* | -2,94 | 2,21E-27 |
| *VC_RS13475* | -2,93 | 4,46E-19 |
| *VC_RS04335* | -2,90 | 6,37E-19 |
| *nrdG* | -2,86 | 8,31E-09 |
| *ompW* | -2,82 | 1,14E-12 |
| *VC_RS14490* | -2,81 | 4,01E-02 |
| *pfkA* | -2,80 | 1,13E-25 |
| *menB* | -2,69 | 5,30E-14 |
| *VC_RS16905* | -2,69 | 5,23E-27 |
| *VC_RS16240* | -2,65 | 1,77E-14 |
| *pflB* | -2,55 | 2,04E-18 |
| *VC_RS10735* | -2,52 | 3,50E-09 |
| *VC_RS02445* | -2,44 | 3,57E-12 |
| *VC_RS10000* | -2,42 | 7,69E-10 |
| *vesC* | -2,41 | 4,62E-15 |
| *VC_RS14330* | -2,41 | 5,14E-15 |
| *VC_RS10325* | -2,41 | 6,01E-11 |
| *gap* | -2,38 | 1,72E-13 |
| *VC_RS03835* | -2,37 | 3,58E-08 |
| *pgi* | -2,30 | 3,04E-18 |
| *VC_RS09625* | -2,30 | 4,67E-14 |
| *malT* | -2,28 | 1,93E-13 |
| *malQ* | -2,26 | 4,59E-15 |
| *VC_RS02160* | -2,20 | 1,11E-14 |
| *VC_RS05160* | -2,19 | 1,61E-17 |
| *VC_RS14510* | -2,19 | 4,33E-04 |
| *VC_RS02215* | -2,18 | 1,52E-05 |
| *VC_RS17420* | -2,18 | 8,35E-17 |
| *glmS* | -2,18 | 3,77E-04 |
| *VC_RS14455* | -2,16 | 7,21E-21 |
| *bioD* | -2,15 | 2,32E-15 |
| *malF* | -2,11 | 7,13E-15 |
| *VC_RS06340* | -2,07 | 2,48E-11 |
| *VC_RS08370* | -2,05 | 1,61E-03 |
| *VC_RS02435* | -2,04 | 7,63E-04 |
| *ppc* | -2,04 | 4,69E-17 |
| *VC_RS12995* | -2,04 | 5,80E-09 |
| *ilvC* | -2,01 | 6,88E-10 |
| *gpmM* | -2,01 | 2,13E-13 |
| *nhaC* | -2,01 | 5,45E-17 |
| *narQ* | -2,00 | 7,24E-04 |
| *feoB* | -1,95 | 1,14E-04 |
| *VC_RS03315* | -1,94 | 9,33E-10 |
| *VC_RS14530* | -1,94 | 1,56E-02 |
| *malK* | -1,93 | 4,62E-15 |
| *VC_RS03320* | -1,92 | 3,28E-06 |
| *raiA* | -1,91 | 6,39E-05 |
| *VC_RS08805* | -1,90 | 2,13E-02 |
| *malE* | -1,90 | 1,05E-13 |
| *VC_RS13960* | -1,89 | 2,50E-04 |
| *VC_RS15980* | -1,89 | 2,11E-11 |
| *eno* | -1,89 | 3,01E-12 |
| *VC_RS14460* | -1,89 | 4,21E-12 |
| *edd* | -1,88 | 7,92E-09 |
| *VC_RS01665* | -1,88 | 8,78E-06 |
| *elbB* | -1,88 | 1,66E-05 |
| *malG* | -1,88 | 6,61E-16 |
| *tpiA* | -1,87 | 2,63E-10 |
| *VC_RS05825* | -1,85 | 4,69E-07 |
| *VC_RS05625* | -1,84 | 5,26E-07 |
| *VC_RS00575* | -1,76 | 3,28E-04 |
| *VC_RS04300* | -1,75 | 9,63E-13 |
| *VC_RS08210* | -1,73 | 1,90E-05 |
| *VC_RS11965* | -1,71 | 1,10E-07 |
| *VC_RS05975* | -1,68 | 4,73E-05 |
| *ptsI* | -1,68 | 2,67E-08 |
| *VC_RS11420* | -1,63 | 1,01E-05 |
| *VC_RS02480* | -1,59 | 4,28E-03 |
| *VC_RS18185* | -1,58 | 7,43E-08 |
| *folA* | -1,58 | 2,16E-05 |
| *VC_RS09155* | -1,57 | 4,41E-05 |
| *VC_RS06100* | -1,56 | 7,43E-03 |
| *VC_RS13010* | -1,55 | 6,39E-03 |
| *tcpP* | -1,55 | 2,45E-06 |
| *glgB* | -1,55 | 3,74E-05 |
| *VC_RS00620* | -1,55 | 6,37E-09 |
| *VC_RS16100* | -1,53 | 1,92E-10 |
| *fbaA* | -1,52 | 1,93E-06 |
| *VC_RS11495* | -1,52 | 3,89E-04 |
| *ushA* | -1,51 | 6,73E-07 |
| *hlyA* | -1,49 | 4,31E-08 |
| *fdh3B* | -1,49 | 5,16E-06 |
| *VC_RS05670* | -1,49 | 1,67E-04 |
| *crp* | -1,47 | 5,77E-10 |
| *VC_RS07315* | -1,46 | 4,15E-09 |
| *VC_RS04785* | -1,46 | 2,40E-07 |
| *nagK* | -1,44 | 6,82E-06 |
| *lamB* | -1,43 | 7,42E-06 |
| *pal* | -1,43 | 2,31E-04 |
| *VC_RS02505* | -1,42 | 2,01E-03 |
| *VC_RS08795* | -1,41 | 8,87E-03 |
| *ccmD* | -1,40 | 4,93E-02 |
| *gltB* | -1,39 | 7,58E-08 |
| *gltB* | -1,39 | 7,58E-08 |
| *VC_RS11435* | -1,38 | 3,62E-05 |
| *VC_RS02090* | -1,38 | 1,06E-02 |
| *fruA* | -1,37 | 3,76E-10 |
| *VC_RS14360* | -1,37 | 6,67E-06 |
| *hcp-2* | -1,37 | 1,35E-03 |
| *hcp-2* | -1,37 | 1,35E-03 |
| *fruB* | -1,36 | 1,89E-07 |
| *VC_RS11635* | -1,36 | 1,17E-05 |
| *VC_RS16670* | -1,35 | 3,55E-04 |
| *VC_RS07305* | -1,35 | 2,69E-06 |
| *VC_RS13950* | -1,34 | 1,50E-03 |
| *VC_RS08520* | -1,34 | 2,22E-05 |
| *menH* | -1,33 | 9,08E-03 |
| *VC_RS05665* | -1,33 | 2,65E-03 |
| *VC_RS17105* | -1,32 | 1,71E-03 |
| *VC_RS02210* | -1,32 | 3,87E-04 |
| *ptsG* | -1,29 | 2,17E-06 |
| *VC_RS05295* | -1,28 | 1,39E-04 |
| *VC_RS01370* | -1,27 | 1,44E-03 |
| *pfkB* | -1,27 | 2,74E-05 |
| *VC_RS09325* | -1,27 | 4,85E-02 |
| *VC_RS02820* | -1,25 | 2,75E-02 |
| *galK* | -1,24 | 3,60E-04 |
| *VC_RS01625* | -1,24 | 2,75E-03 |
| *VC_RS03680* | -1,22 | 2,37E-02 |
| *VC_RS18380* | -1,21 | 1,44E-03 |
| *katG* | -1,21 | 1,44E-03 |
| *pntB* | -1,21 | 1,32E-04 |
| *VC_RS07330* | -1,21 | 4,84E-06 |
| *VC_RS14215* | -1,21 | 1,92E-02 |
| *ccoO* | -1,20 | 4,76E-02 |
| *VC_RS01395* | -1,20 | 2,02E-02 |
| *tssM* | -1,19 | 6,95E-04 |
| *VC_RS01300* | -1,19 | 1,01E-02 |
| *asnB* | -1,18 | 3,77E-05 |
| *tusB* | -1,18 | 2,69E-03 |
| *VC_RS05860* | -1,18 | 4,25E-03 |
| *VC_RS14825* | -1,18 | 9,82E-03 |
| *VC_RS11350* | -1,18 | 2,09E-03 |
| *VC_RS02830* | -1,18 | 3,88E-03 |
| *VC_RS14815* | -1,17 | 2,31E-04 |
| *VC_RS18835* | -1,17 | 3,09E-02 |
| *VC_RS08225* | -1,17 | 2,91E-03 |
| *modC* | -1,16 | 3,88E-03 |
| *VC_RS16360* | -1,16 | 4,86E-03 |
| *VC_RS01390* | -1,16 | 1,96E-04 |
| *VC_RS14820* | -1,15 | 3,49E-04 |
| *tcpH* | -1,15 | 2,13E-02 |
| *VC_RS01790* | -1,14 | 2,16E-05 |
| *VC_RS08240* | -1,14 | 1,35E-02 |
| *tagO* | -1,14 | 4,81E-02 |
| *fliN* | -1,14 | 4,99E-05 |
| *VC_RS05660* | -1,12 | 9,93E-03 |
| *VC_RS00855* | -1,12 | 2,41E-04 |
| *arcA* | -1,12 | 9,12E-03 |
| *arcA* | -1,12 | 9,12E-03 |
| *VC_RS18700* | -1,11 | 4,29E-03 |
| *VC_RS00635* | -1,11 | 3,88E-03 |
| *can* | -1,11 | 1,35E-03 |
| *VC_RS09920* | -1,11 | 7,19E-05 |
| *VC_RS11900* | -1,10 | 1,07E-03 |
| *ycfP* | -1,09 | 1,73E-03 |
| *galM* | -1,08 | 9,41E-03 |
| *VC_RS17495* | -1,08 | 4,37E-05 |
| *VC_RS09885* | -1,07 | 7,65E-04 |
| *VC_RS12600* | -1,07 | 2,42E-02 |
| *VC_RS10600* | -1,07 | 3,60E-04 |
| *VC_RS09255* | -1,06 | 2,13E-02 |
| *VC_RS11255* | -1,06 | 2,99E-03 |
| *oadA* | -1,06 | 1,00E-02 |
| *oadA* | -1,06 | 1,00E-02 |
| *nudF* | -1,05 | 2,57E-03 |
| *VC_RS06640* | -1,05 | 6,18E-04 |
| *VC_RS15915* | -1,05 | 6,89E-04 |
| *rhtB* | -1,04 | 1,80E-02 |
| *rimI* | -1,04 | 3,82E-02 |
| *minE* | -1,04 | 1,57E-02 |
| *VC_RS17175* | -1,04 | 4,67E-04 |
| *VC_RS00770* | -1,03 | 7,83E-03 |
| *VC_RS12105* | -1,03 | 1,93E-02 |
| *hupA* | -1,03 | 2,50E-03 |
| *VC_RS06960* | -1,02 | 3,49E-02 |
| *VC_RS10580* | -1,02 | 6,78E-05 |
| *VC_RS10825* | -1,02 | 4,93E-02 |
| *VC_RS11450* | -1,01 | 1,11E-02 |
| *VC_RS03100* | -1,01 | 8,64E-03 |
| *chiS* | -1,00 | 7,43E-04 |
