# Supplementary material for: Aminoglycoside tolerance in Vibrio cholerae engages translational reprogramming associated with queuosine tRNA modification
Source: eLife. 2025 Jan 6;13:RP96317. doi: 10.7554/eLife.96317 (PMC11703503; doi:10.7554/eLife.96317)
Supplement: Supplementary file 3. [file elife-96317-supp3.docx]

**Supplementary File 3. RNA-seq V. cholerae WT/∆tgt, in MH and TOB. Only significant differences higher than 2-fold are shown.**

| MH |  |  |  |  |  |
| --- | --- | --- | --- | --- | --- |
| locus_tag | **old_locus_tag** | **gene** | **baseMean** | **log2FC WT/∆tgt** | **padj** |
| VC_RS03715 | VC0741,VC_0741 | tgt | 3412 | 6,91 | 3,3E-168 |
| VC_RS13030 | VC2706,VC_2706 | yhhQ | 5207 | 5,12 | 4,6E-148 |
| VC_RS17680 | VC_A0913,VCA0913 | hutB | 298 | 3,23 | 1,1E-06 |
| VC_RS17670 | VC_A0911,VCA0911 | exbB | 234 | 3,18 | 5,8E-07 |
| VC_RS17690 | VC_A0915,VCA0915 | hutD | 131 | 2,91 | 3,5E-05 |
| VC_RS17675 | VC_A0912,VCA0912 | exbD | 329 | 2,88 | 1,6E-09 |
| VC_RS17685 | VC_A0914,VCA0914 | btuC/fecCD | 362 | 2,80 | 2,7E-05 |
| VC_RS03875 | VC0773,VC_0773 | entC | 64 | 2,53 | 2,4E-04 |
| VC_RS16165 | VC_A0576,VCA0576 | hutA | 4750 | 2,51 | 5,1E-06 |
| VC_RS17665 | VC_A0910,VCA0910 | tonB1 | 400 | 2,46 | 9,5E-07 |
| VC_RS14430 | VC_A0229,VCA0229 | febD | 356 | 2,44 | 1,4E-07 |
| VC_RS17655 | VC_A0908,VCA0908 | hutX | 1037 | 2,37 | 9,5E-07 |
| VC_RS14435 | VC_A0230,VCA0230 | fhuC | 617 | 2,34 | 1,7E-04 |
| VC_RS17660 | VC_A0909,VCA0909 | hutW | 1132 | 2,23 | 7,3E-07 |
| VC_RS02435 | VC0475,VC_0475 | cirA | 826 | 2,02 | 1,7E-03 |
| VC_RS14425 | VC_A0228,VCA0228 | fepD | 454 | 2,02 | 1,9E-07 |
| VC_RS17650 | VC_A0907,VCA0907 | hutZ | 3146 | 1,87 | 8,8E-06 |
| VC_RS17955 | VC_A0977,VCA0977 | yejF | 454 | 1,81 | 3,9E-08 |
| VC_RS14420 | VC_A0227,VCA0227 | gcvH | 2378 | 1,78 | 1,4E-05 |
| VC_RS17950 | VC_A0976,VCA0976 |  | 103 | 1,70 | 2,7E-02 |
| VC_RS08170 | VC1688,VC_1688 | mglC | 137 | 1,65 | 1,2E-02 |
| VC_RS07460 | VC1542,VC_1542 | ligA | 54 | 1,64 | 1,3E-04 |
| VC_RS03070 | VC0606,VC_0606 | glnK | 174 | 1,59 | 7,0E-03 |
| VC_RS07475 | VC1545,VC_1545 | exbB | 306 | 1,54 | 1,3E-04 |
| VC_RS07470 | VC1544,VC_1544 | exbD | 354 | 1,46 | 4,2E-04 |
| VC_RS16600 | VC_A0676,VCA0676 | napF | 1952 | 1,45 | 4,1E-02 |
| VC_RS13690 | VC_A0064,VCA0064 | thiS | 198 | 1,44 | 1,8E-03 |
| VC_RS13695 | VC_A0065,VCA0065 | thiG | 180 | 1,42 | 6,3E-04 |
| VC_RS01835 | VC0365,VC_0365 | bfr | 2422 | 1,39 | 1,1E-06 |
| VC_RS07465 | VC1543,VC_1543 |  | 1162 | 1,39 | 3,2E-04 |
| VC_RS10685 | VC2210,VC_2210 | viuB | 550 | 1,34 | 8,9E-04 |
| VC_RS02430 | VC0474,VC_0474 | irgB | 157 | 1,33 | 1,8E-02 |
| VC_RS07595 | VC1572,VC_1572 |  | 27 | 1,28 | 2,9E-02 |
| VC_RS00985 | VC0201,VC_0201 | fhuC | 92 | 1,27 | 3,2E-02 |
| VC_RS00980 | VC0200,VC_0200 | fhuA | 2454 | 1,25 | 3,7E-03 |
| VC_RS07600 | VC1573,VC_1573 | fumC | 197 | 1,25 | 9,7E-05 |
| VC_RS10690 | VC2211,VC_2211 | viuA | 527 | 1,24 | 4,5E-05 |
| VC_RS07480 | VC1546,VC_1546 | exbB | 303 | 1,23 | 2,0E-03 |
| VC_RS03900 | VC0778,VC_0778 | fepG | 53 | 1,19 | 4,6E-02 |
| VC_RS03080 | VC0608,VC_0608 | fbpA | 4516 | 1,18 | 1,3E-04 |
| VC_RS13685 | VC_A0063,VCA0063 | ptrB | 280 | 1,16 | 5,1E-03 |
| VC_RS04975 | VC1009,VC_1009 |  | 505 | 1,16 | 1,1E-05 |
| VC_RS02570 | VC0504,VC_0504 | susC (tonB-like) | 42 | 1,11 | 3,2E-02 |
| VC_RS06170 | VC1265,VC_1265 | cytochrome C | 511 | 1,10 | 1,3E-02 |
| VC_RS07485 | VC1547,VC_1547 | exbB | 692 | 1,09 | 6,0E-04 |
| VC_RS05755 | VC1174,VC_1174 | trpE | 263 | 1,07 | 9,7E-05 |
| VC_RS03865 | VC0771,VC_0771 | vibB | 412 | 1,06 | 1,1E-03 |
| VC_RS16595 | VC_A0675,VCA0675 | narQ | 770 | 1,03 | 4,9E-02 |
| VC_RS16075 | VC_A0558,VCA0558 | yfjD | 516 | 1,03 | 2,5E-02 |
| VC_RS01830 | VC0364,VC_0364 | bfd | 321 | 1,02 | 8,2E-03 |
| VC_RS14440 | VC_A0231,VCA0231 | yqhC | 272 | 1,00 | 2,7E-02 |
| VC_RS02480 | VC0486,VC_0486 | srlR | 748 | 1 | 3,2E-03 |
| VC_RS12970 | VC2694,VC_2694 | sodA | 503 | 0,80 | 5,1E-03 |
|  |  |  |  |  |  |
| TOB |  |  |  |  |  |
| locus_tag | **old_locus_tag** | **gene** | **baseMean** | **log2FoldChange WT/∆tgt** | **padj** |
| VC_RS03715 | VC0741,VC_0741 | tgt | 3412 | 5,991 | 5,0E-181 |
| VC_RS13030 | VC2706,VC_2706 | yhhQ | 5207 | 4,392 | 6,2E-114 |
| VC_RS02575 | VC0505,VC_0505 |  | 8 | 3,806 | 2,8E-02 |
| VC_RS00075 | VC0018,VC_0018 | ibpA | 4235 | 1,919 | 1,5E-03 |
| VC_RS04265 | VC0855,VC_0855 | dnaK | 17310 | 1,863 | 3,5E-03 |
| VC_RS02570 | VC0504,VC_0504 | susC (tonB) | 42 | 1,823 | 2,1E-04 |
| VC_RS04385 | VC0885,VC_0885 |  | 97 | 1,812 | 1,7E-03 |
| VC_RS03575 | VC0711,VC_0711 | clpB | 2525 | 1,688 | 1,2E-02 |
| VC_RS04835 | VC0977,VC_0977 | cnoX | 522 | 1,595 | 2,3E-03 |
| VC_RS02995 | VC0589,VC_0589 | yadG | 518 | 1,586 | 2,3E-02 |
| VC_RS12835 | VC2665,VC_2665 | groES1 | 2421 | 1,555 | 4,6E-02 |
| VC_RS18020 | VC_A0989,VCA0989 | dinF | 136 | 1,511 | 2,5E-02 |
| VC_RS04870 | VC0985,VC_0985 | htpG | 9334 | 1,507 | 4,5E-02 |
| VC_RS05980 | VC1217,VC_1217 | yjgM | 88 | 1,406 | 7,7E-03 |
| VC_RS04390 | VC0886,VC_0886 |  | 208 | 1,381 | 5,8E-03 |
| VC_RS16915 | VC_A0744,VCA0744 | glpK | 1194 | 1,317 | 1,8E-09 |
| VC_RS12880 | VC2674,VC_2674 | hslU | 1673 | 1,268 | 1,6E-02 |
| VC_RS12885 | VC2675,VC_2675 | hslV | 365 | 1,261 | 5,9E-03 |
| VC_RS00930 | VC0188,VC_0188 | prlC | 2248 | 1,194 | 8,4E-03 |
| VC_RS01320 | VC0271,VC_0271 | corC | 728 | 1,155 | 1,2E-03 |
| VC_RS02400 | VC0468,VC_0468 | gshB | 1377 | 1,124 | 1,8E-04 |
| VC_RS06455 | VC1325,VC_1325 | mglB | 1478 | 1,089 | 8,0E-05 |
| VC_RS09260 | VC1920,VC_1920 | lon | 3601 | 1,066 | 2,3E-02 |
| VC_RS02395 | VC0467,VC_0467 | ygqE | 541 | 1,055 | 6,7E-04 |
| VC_RS17520 | VC_A0881,VCA0881 |  | 196 | 1,033 | 1,4E-02 |
| VC_RS17525 | VC_A0882,VCA0882 |  | 289 | 1,021 | 3,2E-03 |
| VC_RS12360 | VC2564,VC_2564 | dbpA | 618 | -1,017 | 3,0E-02 |
| VC_RS15790 | VC_A0494,VCA0494 | acetyltransferase | 70 | -1,028 | 3,7E-02 |
| VC_RS03960 | VC0792,VC_0792 | yjfF oad | 28 | -1,029 | 2,6E-02 |
| VC_RS14205 | VC_A0179,VCA0179 | psuT | 156 | -1,038 | 3,3E-02 |
| VC_RS18615 | VC_1646 |  | 27 | -1,057 | 4,6E-02 |
| VC_RS05270 | VC1071,VC_1071 | arsJ | 117 | -1,093 | 4,0E-03 |
| VC_RS07060 | VC1458,VC_1458 | zot | 640 | -1,102 | 1,8E-02 |
| VC_RS09405 | VC1953,VC_1953 | nupX | 95 | -1,131 | 2,3E-02 |
| VC_RS18070 | VC_A1000,VCA1000 | leuE | 62 | -1,139 | 3,8E-02 |
| VC_RS12345 | VC2561,VC_2561 | cobA | 78 | -1,143 | 3,3E-02 |
| VC_RS15320 |  |  | 90 | -1,171 | 1,4E-02 |
| VC_RS06240 | VC1279,VC_1279 | betT | 572 | -1,246 | 9,9E-04 |
| VC_RS15780 | VC_A0492,VCA0492 | RfbP-related protein | 323 | -1,262 | 8,5E-07 |
| VC_RS07075 | VC1461,VC_1461 | cep ctx | 2195 | -1,282 | 1,9E-04 |
| VC_RS12540 | VC2600,VC_2600 | yejM | 397 | -1,31 | 3,0E-03 |
| VC_RS01360 | VC0280,VC_0280 | cadB | 47 | -1,346 | 5,1E-03 |
| VC_RS16860 | VC_A0732,VCA0732 | ygiW | 881 | -1,417 | 1,5E-03 |
| VC_RS18060 | VC_A0998,VCA0998 | nemA | 385 | -1,446 | 3,0E-02 |
| VC_RS08700 | VC1801,VC_1801 |  | 74 | -1,462 | 2,6E-02 |
| VC_RS01365 | VC0281,VC_0281 | cadA ldcI | 60 | -1,489 | 1,7E-03 |
| VC_RS07640 | VC1581,VC_1581 | nuoL | 56 | -1,502 | 5,4E-03 |
| VC_RS18100 | VC_A1006,VCA1006 | osmC | 77 | -1,521 | 2,0E-03 |
| VC_RS07080 | VC1462,VC_1462 | rstB2 | 4846 | -1,523 | 1,2E-04 |
| VC_RS17365 | VC_A0847,VCA0847 | yjeH | 141 | -1,535 | 3,6E-04 |
| VC_RS05275 | VC1073,VC_1073 |  | 235 | -1,617 | 1,0E-05 |
| VC_RS08705 | VC1802,VC_1802 |  | 35 | -1,77 | 8,5E-03 |
| VC_RS08695 |  |  | 66 | -1,778 | 1,3E-03 |
| VC_RS08680 | VC1798,VC_1798 | eha | 131 | -1,804 | 2,2E-05 |
| VC_RS08690 | VC1800,VC_1800 |  | 183 | -1,851 | 8,0E-05 |
| VC_RS07065 | VC1459,VC_1459 | ace | 152 | -1,939 | 4,0E-02 |
| VC_RS08685 | VC1799,VC_1799 | phage transposase | 302 | -2,376 | 1,4E-08 |
| VC_RS07040 | VC1454,VC_1454 | rstA1 | 29530 | -2,705 | 1,9E-04 |
| VC_RS07070 | VC1460,VC_1460 | orfU ctx | 1156 | -2,774 | 3,9E-08 |
| VC_RS07085 | VC1463,VC_1463 | rstA2 | 29040 | -2,893 | 5,1E-05 |
