# Supplementary material for: Aminoglycoside tolerance in Vibrio cholerae engages translational reprogramming associated with queuosine tRNA modification
Source: eLife. 2025 Jan 6;13:RP96317. doi: 10.7554/eLife.96317 (PMC11703503; doi:10.7554/eLife.96317)
Supplement: Supplementary file 4. [file elife-96317-supp4.docx]

**Supplementary File 4: Primers, plasmids and strains**

| gene expressions from pSEVA | | | |
| --- | --- | --- | --- |
| plasmid in strain TOP10 | name | | primers used for gene amplification |
| M027 | pSEVA238 | | MCSSEVA238-5 GCAAGAAGCGGATACAGGAG  MCSSEVA238-3 GGTTTTCCCAGTCACGACGC |
| R591 | pSEVA-tgt | | 5tgtEcoRI CGCGGAATTCGTGAAATTAAAATTTGAACTG and  3tgtXbaI CGCGTCTAGATCAGGCTTTGTCTTTTTGTAGTGG |
| Q298 | pSEVA-rsxA | | ZIP753 GCCCGAATTCCATTTGCCACTTATTGCG and  ZIP754 GCCGCCGAATTCTTACAGTTTCACCAATCCGGTAAAGCC |
| Q294 | pSEVA-soxR | | ZIP757 GGGGCCCCCCCCGAATTCGTAAAGCGTTTTTTTAATAAAACGGG and ZIP758 CCGAATTCTTAACGGCTCCACTCTTCTGGATGCGATAAGCG |
| Q291 | pSEVA-katG | | ZIP755 GCGCGCCCCGAATTCATTTCCCATTTAGTGAAAAGGG and  ZIP756 GAATTCTTACATCGCGGCCAGTTTTGCCACC |
| tRNA overexpression fragments cloned in pTOPO-blunt-kanamycin R (Ptrc promoter and VCt002 terminator are underlined, anticodon in red) | | | |
| plasmid in TOP10 | name | | sequence |
| L961 | Ptrc-eco-tRNA-Tyr wtGUA | | GAGCTGTTGACAATTAATCATCCGGCTCGTATAATGTGTGGGGTGGGGTTCCCGAGCGGCCAAAGGGAGCAGACTGTAAATCTGCCGTCACAGACTTCGAAGGTTCGAATCCTTCCCCCACCACCACTTATTCGAGCTTAAGCTCAAAAAACTACA |
| M660 | Ptrc-eco-tRNA-Tyr mutAUA | | GAGCTGTTGACAATTAATCATCCGGCTCGTATAATGTGTGGGGTGGGGTTCCCGAGCGGCCAAAGGGAGCAGACTATAAATCTGCCGTCACAGACTTCGAAGGTTCGAATCCTTCCCCCACCACCACTTATTCGAGCTTAAGCTCAAAAAACTACA |
| L957 | Ptrc-eco-tRNA-Asp wt GUC | | GAGCTGTTGACAATTAATCATCCGGCTCGTATAATGTGTGGGGAGCGGTAGTTCAGTCGGTTAGAATACCTGCCTGTCACGCAGGGGGTCGCGGGTTCGAGTCCCGTCCGTTCCGCCACTTATTCGAGCTTAAGCTCAAAAAACTACA |
| L960 | Ptrc-vch-tRNA-Tyr wtGUA | | GAGCTGTTGACAATTAATCATCCGGCTCGTATAATGTGTGGGGAGGGGTTCCCGAGTGGCCAAAGGGAGCAGACTGTAAATCTGCCGGCTCCGCCTTCGATGGTTCGAATCCGTCCCCCTCCACCACTTATTCGAGCTTAAGCTCAAAAAACTACA |
| L959 | Ptrc-vch-tRNA-Tyr mutAUA | | GAGCTGTTGACAATTAATCATCCGGCTCGTATAATGTGTGGGGAGGGGTTCCCGAGTGGCCAAAGGGAGCAGACTATAAATCTGCCGGCTCCGCCTTCGATGGTTCGAATCCGTCCCCCTCCACCACTTATTCGAGCTTAAGCTCAAAAAACTACA |
| L956 | Ptrc-vch-tRNA-Asp wtGUC | | GAGCTGTTGACAATTAATCATCCGGCTCGTATAATGTGTGGGGAGCGGTAGTTCAGTCGGTTAGAATACCGGCCTGTCACGCCGGGGGTCGCGGGTTCGAGTCCCGTCCGCTCCGCCACTTATTCGAGCTTAAGCTCAAAAAACTACA |
| L955 | Ptrc-vch-tRNA- mutAspAUC | | GAGCTGTTGACAATTAATCATCCGGCTCGTATAATGTGTGGGGAGCGGTAGTTCAGTCGGTTAGAATACCGGCCTATCACGCCGGGGGTCGCGGGTTCGAGTCCCGTCCGCTCCGCCACTTATTCGAGCTTAAGCTCAAAAAACTACA |
| M651 | Ptrc-vch-tRNA-Asn wtGUU | | GAGCTGTTGACAATTAATCATCCGGCTCGTATAATGTGTGGTCCTCCTTAGCTCAGTCGGTAGAGCGACGGACTGTTAATCCGCAGGTCGCTGGTTCAAGTCCAGCAGGAGGAGCCACTTATTCGAGCTTAAGCTCAAAAAACTACA |
| L962 | Ptrc-vch-tRNA-Asn mutAUU | | GAGCTGTTGACAATTAATCATCCGGCTCGTATAATGTGTGGTCCTCCTTAGCTCAGTCGGTAGAGCGACGGACTATTAATCCGCAGGTCGCTGGTTCAAGTCCAGCAGGAGGAGCCACTTATTCGAGCTTAAGCTCAAAAAACTACA |
| M646 | Ptrc-vch-tRNA-HIS wtGUG | | GAGCTGTTGACAATTAATCATCCGGCTCGTATAATGTGTGGGTGGCTATAGCTCAGTTGGTAGAGCCCCGGATTGTGATTCCGGTTGTCGTGGGTTCGAGCCCCATTAGCCACCCCACTTATTCGAGCTTAAGCTCAAAAAACTACA |
| L997 | Ptrc-vch-tRNA-HIS mutAUG | | GAGCTGTTGACAATTAATCATCCGGCTCGTATAATGTGTGGGTGGCTATAGCTCAGTTGGTAGAGCCCCGGATTATGATTCCGGTTGTCGTGGGTTCGAGCCCCATTAGCCACCCCACTTATTCGAGCTTAAGCTCAAAAAACTACA |
| M653 | Ptrc-vch-tRNA-Phe wtGAA | | GAGCTGTTGACAATTAATCATCCGGCTCGTATAATGTGTGGGCCCGGATAGCTCAGTCGGTAGAGCAGAGGATTGAAAATCCTCGTGTCGGTGGTTCGATTCCGCCTCCGGGCACCACTTATTCGAGCTTAAGCTCAAAAAACTACA |
| translational fusions ordered in pUC_IDT (carbenicillin R) | | | |
| plasmid in strain DH5α | name | | sequence |
| R973 | Ptrc-rsxATAC-gfp | | TTGACAATTAATCATCCGGCTCGTATAATGTGTGGAATTGTGAGCGGATAACAATTTCACACAGGAAACAGCGCCGCATGACCGAATACCTTTTGTTGTTAATCGGCACCGTGCTGGTCAATAACTTTGTACTGGTGAAGTTTTTGGGCTTATGTCCTTTTATGGGCGTATCAAAAAAACTAGAGACCGCCATTGGCATGGGGTTGGCGACGACATTCGTCCTCACCTTAGCTTCGGTGTGCGCTTACCTGGTGGAAAGTTACGTGTTACGTCCGCTCGGCATTGAGTACCTGCGCACCATGAGCTTTATTTTGGTGATCGCTGTCGTAGTACAGTTCACCGAAATGGTGGTGCACAAAACCAGTCCGACACTCTACCGCCTGCTGGGCATTTTCCTGCCACTCATCACCACCAACTGTGCGGTATTAGGGGTTGCGCTGCTCAACATCAACGAAAATCACAACTTTATTCAATCGATCATTTACGGTTTTGGCGCTGCTGTTGGCTTCTCGCTGGTGCTCATCTTGTTCGCTTCAATGCGTGAGCGAATCCATGTAGCCGATGTCCCCGCTCCCTTTAAGGGCGCATCCATTGCGATGATCACCGCAGGTTTAATGTCTTTGGCCTTTATGGGCTTTACCGGATTGGTGAAACTGGCTAGCAAAGGAGAAGAACTTTTCACTGGAGTTGTCCCAATTCTTGTTGAATTAGATGGTGATGTTAATGGGCACAAATTTTCTGTCAGTGGAGAGGGTGAAGGTGATGCTACATACGGAAAGCTTACCCTTAAATTTATTTGCACTACTGGAAAACTACCTGTTCCATGGCCAACACTTGTCACTACTTTGACCTATGGTGTTCAATGCTTTTCCCGTTATCCGGATCATATGAAACGGCATGACTTTTTCAAGAGTGCCATGCCCGAAGGTTATGTACAGGAACGCACTATATCTTTCAAAGATGACGGGAACTACAAGACGCGTGCTGAAGTCAAGTTTGAAGGTGATACCCTTGTTAATCGTATCGAGTTAAAAGGTATTGATTTTAAAGAAGATGGAAACATTCTCGGACACAAACTCGAGTACAACTATAACTCACACAATGTATACATCACGGCAGACAAACAAAAGAATGGAATCAAAGCTAACTTCAAAATTCGCCACAACATTGAAGATGGAGGCGTTCAACTAGCAGACCATTATCAACAAAATACTCCAATTGGCGATGGCCCTGTCCTTTTACCAGACAACCATTACCTGTCGACACAATCTGCCCTTTCGAAAGATCCCAACGAAAAGCGTGACCACATGGTCCTTCTTGAGTTTGTAACTGCTGCTGGGATTACACATGGCATGGATGAGCTCTACAAATAA |
| R972 | Ptrc-rsxATATWT-gfp | | TTGACAATTAATCATCCGGCTCGTATAATGTGTGGAATTGTGAGCGGATAACAATTTCACACAGGAAACAGCGCCGCATGACCGAATATCTTTTGTTGTTAATCGGCACCGTGCTGGTCAATAACTTTGTACTGGTGAAGTTTTTGGGCTTATGTCCTTTTATGGGCGTATCAAAAAAACTAGAGACCGCCATTGGCATGGGGTTGGCGACGACATTCGTCCTCACCTTAGCTTCGGTGTGCGCTTATCTGGTGGAAAGTTACGTGTTACGTCCGCTCGGCATTGAGTATCTGCGCACCATGAGCTTTATTTTGGTGATCGCTGTCGTAGTACAGTTCACCGAAATGGTGGTGCACAAAACCAGTCCGACACTCTATCGCCTGCTGGGCATTTTCCTGCCACTCATCACCACCAACTGTGCGGTATTAGGGGTTGCGCTGCTCAACATCAACGAAAATCACAACTTTATTCAATCGATCATTTATGGTTTTGGCGCTGCTGTTGGCTTCTCGCTGGTGCTCATCTTGTTCGCTTCAATGCGTGAGCGAATCCATGTAGCCGATGTCCCCGCTCCCTTTAAGGGCGCATCCATTGCGATGATCACCGCAGGTTTAATGTCTTTGGCCTTTATGGGCTTTACCGGATTGGTGAAACTGGCTAGCAAAGGAGAAGAACTTTTCACTGGAGTTGTCCCAATTCTTGTTGAATTAGATGGTGATGTTAATGGGCACAAATTTTCTGTCAGTGGAGAGGGTGAAGGTGATGCTACATACGGAAAGCTTACCCTTAAATTTATTTGCACTACTGGAAAACTACCTGTTCCATGGCCAACACTTGTCACTACTTTGACCTATGGTGTTCAATGCTTTTCCCGTTATCCGGATCATATGAAACGGCATGACTTTTTCAAGAGTGCCATGCCCGAAGGTTATGTACAGGAACGCACTATATCTTTCAAAGATGACGGGAACTACAAGACGCGTGCTGAAGTCAAGTTTGAAGGTGATACCCTTGTTAATCGTATCGAGTTAAAAGGTATTGATTTTAAAGAAGATGGAAACATTCTCGGACACAAACTCGAGTACAACTATAACTCACACAATGTATACATCACGGCAGACAAACAAAAGAATGGAATCAAAGCTAACTTCAAAATTCGCCACAACATTGAAGATGGAGGCGTTCAACTAGCAGACCATTATCAACAAAATACTCCAATTGGCGATGGCCCTGTCCTTTTACCAGACAACCATTACCTGTCGACACAATCTGCCCTTTCGAAAGATCCCAACGAAAAGCGTGACCACATGGTCCTTCTTGAGTTTGTAACTGCTGCTGGGATTACACATGGCATGGATGAGCTCTACAAATAA |
| R975 | Ptrc-gfpTAC | | GAGCTGTTGACAATTAATCATCCGGCTCGTATAATGTGTGGAATTGTGAGCGGATAACAATTTCACACAGGAAACACATATGCGTAAAGGAGAAGAACTTTTCACTGGAGTTGTCCCAATTCTTGTTGAATTAGATGGTGATGTTAATGGGCACAAATTTTCTGTCAGTGGAGAGGGTGAAGGTGATGCAACATACGGAAAACTTACCCTTAAATTTATTTGCACTACTGGAAAACTACCTGTTCCATGGCCAACACTTGTCACTACTTTCGGTTACGGTGTTCAATGCTTTGCGAGATACCCAGATCATATGAAACAGCATGACTTTTTCAAGAGTGCCATGCCCGAAGGTTACGTACAGGAAAGAACTATATTTTTCAAAGATGACGGGAACTACAAGACACGTGCTGAAGTCAAGTTTGAAGGTGATACCCTTGTTAATAGAATCGAGTTAAAAGGTATTGATTTTAAAGAAGATGGAAACATTCTTGGACACAAATTGGAATACAACTACAACTCACACAATGTATACATCATGGCAGACAAACAAAAGAATGGAATCAAAGTTAACTTCAAAATTAGACACAACATTGAAGATGGAAGCGTTCAACTAGCAGACCATTACCAACAAAATACTCCAATTGGCGATGGCCCTGTCCTTTTACCAGACAACCATTACCTGTCCACACAATCTGCCCTTTCGAAAGATCCCAACGAAAAGAGAGACCACATGGTCCTTCTTGAGTTTGTAACAGCTGCTGGGATTACACATGGCATGGATGAACTATACAAATAA |
| R974 | Ptrc-gfpTAT | | GAGCTGTTGACAATTAATCATCCGGCTCGTATAATGTGTGGAATTGTGAGCGGATAACAATTTCACACAGGAAACACATATGCGTAAAGGAGAAGAACTTTTCACTGGAGTTGTCCCAATTCTTGTTGAATTAGATGGTGATGTTAATGGGCACAAATTTTCTGTCAGTGGAGAGGGTGAAGGTGATGCAACATATGGAAAACTTACCCTTAAATTTATTTGCACTACTGGAAAACTACCTGTTCCATGGCCAACACTTGTCACTACTTTCGGTTATGGTGTTCAATGCTTTGCGAGATATCCAGATCATATGAAACAGCATGACTTTTTCAAGAGTGCCATGCCCGAAGGTTATGTACAGGAAAGAACTATATTTTTCAAAGATGACGGGAACTATAAGACACGTGCTGAAGTCAAGTTTGAAGGTGATACCCTTGTTAATAGAATCGAGTTAAAAGGTATTGATTTTAAAGAAGATGGAAACATTCTTGGACACAAATTGGAATATAACTATAACTCACACAATGTATATATCATGGCAGACAAACAAAAGAATGGAATCAAAGTTAACTTCAAAATTAGACACAACATTGAAGATGGAAGCGTTCAACTAGCAGACCATTATCAACAAAATACTCCAATTGGCGATGGCCCTGTCCTTTTACCAGACAACCATTATCTGTCCACACAATCTGCCCTTTCGAAAGATCCCAACGAAAAGAGAGACCACATGGTCCTTCTTGAGTTTGTAACAGCTGCTGGGATTACACATGGCATGGATGAACTATATAAATAA |
| plasmid in strain (WT/*∆tgt*) | codon replacement | | introduction of point mutation in circular pTOPO-TA (kanamycin R) by PCR using primers : |
| N509/N511 | bla wt | | Tyr103TAC Asp129GAT |
| N512/N514 | bla Tyr103TAC  > Tyr TAT synonymous | | ZIP555 GACTTGGTTGAGTATTCACCAGTCACAGAAAAGC  ZIP556 TCTGTGACTGGTGAATACTCAACCAAGTCATTCTGAGAATAG |
| plasmid in strain TOP10 | primers for transcriptional fusion PrsxA-gfp | | |
| Q282 | ZIP796  ZIP812  ZIP813  ZIP200 | | GGATACAAAAAAGTAAACCC  CTCCTTTACGCATAGTTATATAAATGTTTGCTTCCGATCCCGGCATTATCCTG  CAGGATAATGCCGGGATCGGAAGCAAACATTTATATAACTATGCGTAAAGGAG  TATCAAGCTTATTTGTATAGTTCATCCATGCC |
| target for amplification | primers for q-RT-PCR tRNAs | | |
| tRNATyr | ZIP719  ZIP720 | | GGAGGGGTTCCCGAGTGG  GGTGGAGGGGGACGGATT |
| tRNAAsp | ZIP721  ZIP722 | | GGAGCGGTAGTTCAGTCG  TGGCGGAGCGGACGGGAC |
| tRNAHis | ZIP723  ZIP724 | | GTGGCTATAGCTCAGTTG  TGGGGTGGCTAATGG |
| tRNAAsn | ZIP725  ZIP726 | | TCCTCCTTAGCTCAGTCGG  TGGCTCCTCCTGCTGG |
| gyrA | gyrA_F  gyrA_R | | AAT GTG CTG GGC AAC GAC TG  GAG CCA AAG TTA CCT TGG CC |
| target for amplification | primers for digital-RT-PCR  fluorescent probes | | |
| *tgt* | | | fwd CAA CAC CAC TGG ATC CTC ATT  rev GGT AGT AAC GCA GGT TAT GG  5’ - [FAM] A CCT GCA TCA TCT GGA TCG CTG TAA - 3’ [BHQ2] |
| *rsxA* | | | fwd TCA CGC ATT GAA GCG AAC  rev CAC CAA CTG TGC GGT ATT AG  5’ - [FAM] A GCG CCA AAA CCA TAA ATG ATC GAT - 3’ [BHQ2] |
| *gyrA* | | | fwd AAT GTG CTG GGC AAC GAC TG  rev GAG CCA AAG TTA CCT TGG CC  5’ - [CY5] - CAC CCT CAT GGT GAC AGT GCG GTT T - 3’ [BHQ2] |
| Strains | | |  |
| *V. cholerae* | | strain # | reference |
| *N16961 hapR+* | | F606 | laboratory collection |
| *N16961 hapR+ ∆lacZ* | | K329 | Babosan et al, 2022 |
| *∆tgt::spec* | | J420 | Babosan et al, 2022 |
| *∆tolA::kan* | | J983 | Negro et al, 2019 |
| *∆crp::kan* | | Q081 | Deletion of *crp* in F606 as described in Lang et al, 2021 |
| *∆rluF::kan* | | M567 | Babosan et al, 2022 |
| *∆rluF::kan ∆tgt::spec* | | M569 | Deletion of *rluF* in J420, as described for deletion of *rluF* in WT in Babosan et al, 2022 |
| *E. coli* | | strain # | construction |
| *MG1655* | | C349 | laboratory collection |
| *∆tgt::kan* | | J233 | P1 transduction of tgt::kan from Keio collection into MG1655 |
| *∆tgt* | | R181 | Kanamycin resistance cassette was removed using the FLP/FRT system (Zhu et al, 1995) |
| *∆rsxA::kan* | | R207 | P1 transduction of tgt::kan from Keio collection into MG1655 |
| *∆tgt ∆rsxA::kan* | | R223 | P1 transduction of rsxA::kan from Keio collection into R181 *∆tgt* |
